# Low-dose aspirin and incidence of lung carcinoma in patients with chronic obstructive pulmonary disease in Hong Kong: A cohort study

**DOI:** 10.1371/journal.pmed.1003880

**Published:** 2022-01-13

**Authors:** Si-Yeung Yu, Mary Sau-Man Ip, Xue Li, Ka-Shing Cheung, Qing-Wen Ren, Mei-Zhen Wu, Hang-Long Li, Pui-Fai Wong, Hung-Fat Tse, Kai-Hang Yiu

**Affiliations:** 1 Cardiology Division, Department of Medicine, The University of Hong Kong-Shen Zhen Hospital, Shenzhen City, Guangdong Province, China; 2 Department of Medicine, The University of Hong Kong, Queen Mary Hospital, Hong Kong, China; Johns Hopkins: Johns Hopkins University, UNITED STATES

## Abstract

**Background:**

Evidence suggests that chronic obstructive pulmonary disease (COPD) is associated with a higher risk of lung carcinoma. Using a territory-wide clinical electronic medical records system, we investigated the association between low-dose aspirin use (≤160 mg) among patients with COPD and incidence of lung carcinoma and the corresponding risk of bleeding.

**Methods and findings:**

This is a retrospective cohort study conducted utilizing Clinical Data Analysis Reporting System (CDARS), a territory-wide database developed by the Hong Kong Hospital Authority. Inverse probability of treatment weighting (IPTW) was used to balance baseline covariates between aspirin nonusers (35,049 patients) with new aspirin users (7,679 patients) among all eligible COPD patients from 2005 to 2018 attending any public hospitals. The median age of the cohort was 75.7 years (SD = 11.5), and 80.3% were male. Competing risk regression with Cox proportional hazards model were performed to estimate the subdistribution hazard ratio (SHR) of lung carcinoma with low-dose aspirin and the associated bleeding events. Of all eligible patients, 1,779 (4.2%, 1,526 and 253 among nonusers and users) were diagnosed with lung carcinoma over a median follow-up period of 2.6 years (interquartile range [IQR]: 1.4 to 4.8). Aspirin use was associated with a 25% lower risk of lung carcinoma (SHR = 0.75, 95% confidence interval [CI] 0.65 to 0.87, *p* = <0.001) and 26% decrease in lung carcinoma–related mortality (SHR = 0.74, 95% CI 0.64 to 0.86, *p* = <0.001). Subgroup analysis revealed that aspirin was beneficial for patients aged above or below 75 years, but was also beneficial among populations who were male, nondiabetic, and nonhypertensive. Aspirin use was not associated with an increased risk of upper gastrointestinal bleeding (UGIB) (SHR = 1.19, 95% CI 0.94 to 1.53, *p* = 0.16), but was associated with an increased risk of hemoptysis (SHR = 1.96, 95% CI 1.73 to 2.23, *p* < 0.001). The main limitations of the study were (i) that one group of patients may be more likely to seek additional medical attention, although this was partially mitigated by the use of propensity score analysis; and (ii) the observational nature of the study renders it unable to establish causality between aspirin use and lung carcinoma incidence.

**Conclusions:**

In this study, we observed that low-dose aspirin use was associated with a lower risk of lung carcinoma and lung carcinoma–related mortality among COPD patients. While aspirin was not associated with an increased risk of UGIB, the risk of hemoptysis was elevated.

## Abstract 2

In a cohort study, Si-Yeung Yu and colleagues investigate the association between low-dose aspirin and incidence of lung carcinoma in patients with chronic obstructive pulmonary disease in Hong Kong.

## Author summary

**Why was this study done?:**

- Lung carcinoma is the most common malignancy worldwide, and chronic obstructive pulmonary disease (COPD), for which no curative treatments exist, is a prevalent risk factor of lung carcinoma.
- Aspirin is a medication with potential anticancer effects. Its effect on lung cancer, however, remains controversial. To our knowledge, its effect in patients in COPD remains unstudied.
- Aspirin is also known to cause bleeding. However, its effects in COPD patients, again to our knowledge, also remains unstudied.

**What did the researchers do and find?:**

- Using a territory-wide database, the risk of lung carcinoma was assessed in patients with COPD defined as aspirin nonusers (35,049) and aspirin users (7,679 patients). A mathematical model was used, which balances the 2 cohorts by their comorbidities, drug uses, demographic variables, and proxy variables for their socioeconomic status.
- Aspirin use was associated with a 25% lower risk of lung carcinoma, implying that within our cohort, every 125 patients treated for 5 years is associated with one fewer case of lung carcinoma.
- We found, additionally, that aspirin use was associated with an increased risk of coughing up blood, although the overall risk of gastrointestinal bleeding is not increased.

**What do these findings mean?:**

- Aspirin may be implicated as a potential preventative treatment for lung carcinoma in COPD patients, but the increased risk of adverse effects merits further evaluation.
- The current study design was unable to eliminate the possibility that aspirin users were more likely to seek additional treatment, although this is mitigated through design choices in developing the mathematical model.
- The observational nature of the study prevents causality from being established.
- The use of aspirin in COPD should depend on the benefits and harms associated with its usage, when they have been thoroughly studied in randomized trials.

## Introduction

Lung carcinoma is the most common cause of malignancy worldwide with an estimated incidence of 2.1 million in 2018, resulting in 1.8 million deaths [1]. Cigarette users, of which there are currently 1 billion worldwide, are especially at risk. In addition, chronic obstructive pulmonary disease (COPD), a progressive airway disease caused by cigarette smoking, is by far the most common comorbidity in patients with lung carcinoma, with a prevalence ranging from 30% to 70% [2]. Among smokers, the risk of lung carcinoma development is higher in patients with COPD than in those without COPD, suggesting that there may be common pathogenic mechanisms between these 2 entities [3]. Given the lack of curative interventions for COPD, preventative strategies for lung carcinoma in patients with COPD is urgently needed.

Prior studies have suggested that aspirin may prevent acute exacerbations, reduce hospitalizations, and improve lung function as well as survival in patients with COPD [4,5]. Further, experimental [6,7] and clinical data [8–10] suggested that aspirin may prevent carcinogenesis in various systems. The postulated mechanism for the protective effect of aspirin includes inhibition of the pro-inflammatory cyclooxygenase enzymes and its antiplatelet properties [11–13]. A recent cohort study involving health screening participants has demonstrated that the use of low-dose aspirin for more than 5 years provides a modest risk reduction of lung carcinoma [14]. However, detailed information of concomitant drug use and individual comorbidities were not available, and these confounding factors have not been adjusted for, which predisposes patients to confounding biases. Meta-analyses pertaining to the association between aspirin and lung carcinoma in the general population has yielded conflicting results [15,16]. Moreover, to the best of our knowledge, the ability of aspirin to reduce lung carcinoma in patients with COPD, a population more susceptible to lung carcinoma, has not been specifically evaluated. Evaluation of the association between aspirin and lung carcinoma in this specific population may be more clinically relevant, not only because the elevated susceptibility to lung carcinoma implies a greater need for chemoprophylaxis, but also because additional biological cascades are up-regulated in COPD, some of which are mediated by the effects of cyclooxygenases, and thus may be targeted by aspirin [12]. In this territory-wide cohort study, we examined the relation between the use of aspirin and incident lung carcinoma and related mortality among patients with COPD. In addition, the risks of upper gastrointestinal bleeding (UGIB) and hemoptysis associated with aspirin use were also studied to evaluate for potential adverse effects.

## Methods

This was a retrospective cohort study conducted with data from the Clinical Data Analysis Reporting System (CDARS), a territory-wide database developed by the Hong Kong Hospital Authority. As the statutory body and the singular provider of public healthcare services in Hong Kong, the Hospital Authority provides over 80% of inpatient services in Hong Kong, a territory with a Chinese-predominant population of 7.5 million. The hospital authority is responsible not only for 42 public hospitals, but also for all 47 public specialist outpatient clinics and 73 general outpatient clinics [17]. CDARS prospectively collects patient information including, but not limited to, demographic data, diagnoses, drug prescriptions, procedures, laboratory tests, and episodes of hospital visits. Many cohort studies have been performed using information from CDARS previously, with data validation showing a high percentage of coding accuracy [17–19]. Personal information (name and Hong Kong identification number) were deidentified in CDARS, and unique reference numbers were generated. The study was approved by the institutional review board (IRB) of the University of Hong Kong and the West Cluster of the Hong Kong Hospital Authority (Reference number UW 20–810), with prespecified outcomes and statistical methods as documented on the IRB application (S1 File). This study is reported as per the REporting of studies Conducted using Observational Routinely collected Data (RECORD) guideline (S2 File) [20].

### Outcome definition and study cohort

We searched for all patients aged 18 or above with COPD who attended any public hospitals between January 1, 2005 and December 31, 2018. We subsequently identified all episodes of aspirin dispenses among the cohort, and the index date for aspirin users were defined as the first date of aspirin prescription. Nonusers were assigned a randomly generated clinical visit date as index date [21,22]. To ensure a new user design, participants must not have received aspirin or other antiplatelets within an entry period prior to the index date lasting 180 days. We also excluded patients who received other antiplatelet medications during a subsequent entry period of 90 days or have history of human immunodeficiency disease, lung carcinoma, or any history of excisional procedures of the lung, including lung transplants before the 90-day entry period ends. We traced patient records on CDARS prior to the index date and collected data pertaining information of covariates including age at index date, sex, comorbidities (diabetes, obesity, hypertension, cerebrovascular diseases, peripheral vascular diseases, congestive heart failure, coronary heart disease, arrhythmias, gastrointestinal bleeding and nongastrointestinal bleeding, cirrhosis, and coagulation defects), and drug history (use of antihypertensives, insulin, antidiabetics, beta-blockers, bronchodilators, insulin, nonsteroidal anti-inflammatory drugs (NSAIDs), lipid-regulating drugs, and inhaled steroids) as well as factors relating to their socioeconomic status (alcoholism, nonsmoking etiologies of COPD, intravenous drug use, and number of inpatient hospital visits in the year prior to index). Prior drug use was defined as ≥90 cumulative defined daily dose (DDD).

The primary outcome of the study was that of newly diagnosed lung carcinoma subsequent to diagnosis of COPD. The follow-up for patients began on index date and lasted until a diagnosis of lung carcinoma, death, excisional procedures, and transplants or of June 30, 2020, whichever is sooner. Furthermore, we looked at whether aspirin use was associated with differing risks of lung-related mortality. Where histological classification of the lung carcinoma was accessible, the hazard ratios for major histological types were reported. In addition, we also looked at whether aspirin affected the odds of a patient receiving a diagnosis of bleeding, as well as the type of bleeding associated with aspirin use. In the case that aspirin was associated with bleeding, we also adjusted for the effect of an assumed discontinuation accordingly when reporting the association between aspirin and lung carcinoma.

### Exposure definition

Our study mimicked an intention-to-treat design, where aspirin exposure was defined as ≥90 consecutive cumulative DDD of aspirin beginning at the index date [18,22]. Patients who received aspirin for a period less than 90 consecutive DDD were excluded [23]. Due to the lack of international standardization of aspirin doses, WHO recommended a dose definition as 1 tablet of aspirin independent of tablet strength [24]. In Hong Kong, aspirin medications are available in formulations of 75 mg, 80 mg, 100 mg, and 300 mg. We used a low-dose aspirin definition with an upper threshold of 160 mg and excluded patients who used an aspirin dosage above the threshold. The DDD was therefore obtained by summing the number of tablets taken by eligible patients. Details of ICD-9 codes used are in S1 Table.

### Main analysis

In order to avoid biases due to confounders for treatment selection due to lack of randomization, an inverse probability of treatment weighting (IPTW) was used. Covariates that were considered prognostically significant as well as those that influence treatment selection were regressed to the probability of receiving treatment [25]. The propensity score model was evaluated by the C-statistic. Upon the application of IPTW, covariates were considered balanced between aspirin users and nonusers if the standardized mean difference is ≤0.10. We utilized a Cox proportional hazards model to determine the association between aspirin use and lung carcinoma [26]. A Fine and Gray model, which accounted for mortality and excisional lung procedures as competing events, was used in analyzing aspirin’s association with lung carcinoma [27]. Using the same method, we investigated the association between aspirin and lung carcinoma–related mortalities as well, with mortality attributed to nonlung carcinoma causes and excisional lung procedures as competing events. To allow for better appreciation of the clinical implications, we derived the number needed to treat (NNT) to prevent a case of lung carcinoma at 3 and 5 years.

To determine the risk of subsequent bleeding events among aspirin users compared to nonusers, we first searched for all diagnoses of bleeding episodes (UGIB and hemoptysis) and analyzed the difference in the number of bleeding events by Poisson regression, adjusting for the aforementioned covariates and taking propensity score weightings into consideration. We also utilized a Cox proportional hazards to determine the hazard ratio of aspirin use on time to first bleeding event, as this model better reflected the association between aspirin and bleeding should there have been aspirin discontinuation upon first instance of bleeding.

### Subgroup and sensitivity analyses

Subgroups were analyzed as stratified by their age, sex, comorbidities, and drug use, and the incidence per person-years was reported alongside the subdistribution hazard ratio (SHR) determined by competing risk regression as well as results of tests for interaction. Several sensitivity analyses were conducted: (1) a conventional cox regression without competing risks or IPTW to allow comparison to previous cohort studies [14,28,29]. (2) A competing risk regression was performed without IPTW, instead including the propensity score as an additional variable in cox regression. (3) The association between aspirin use and lung carcinoma beginning from 4 years after the index date was investigated, to minimize effects of reverse causation. This was done by excluding all patients with outcomes occurring within 4 years. (4) To account for the effect of possible discontinuation of aspirin upon initiation of another antiplatelet, we censored all patients upon initiation of another antiplatelet. (5) We excluded all patients below the age of 40 to limit diagnostic or coding errors. (6) We used an alternative definition of aspirin use, instead defining aspirin use as more than 290 DDD of aspirin use within the first year since index date, i.e., requiring aspirin users to be more than approximately 80% adherent to a daily regimen [22]. (7) The association between aspirin use and lung carcinoma was assessed after excluding cancers with a history of malignant neoplasms, to reduce the number of secondary lung cancers. (8) Since aspirin’s effect may be partially mediated by a lower risk of metastasis [30,31], we also matched aspirin users and nonusers additionally based on a prior history of malignancy. (9) In addition, we reported the E-value that provides information on the strength of unmeasured confounders required to invalidate results [32]. All statistical analyses were performed using R v4.0.3.

## Results

### Patient characteristics

**Fig 1:**
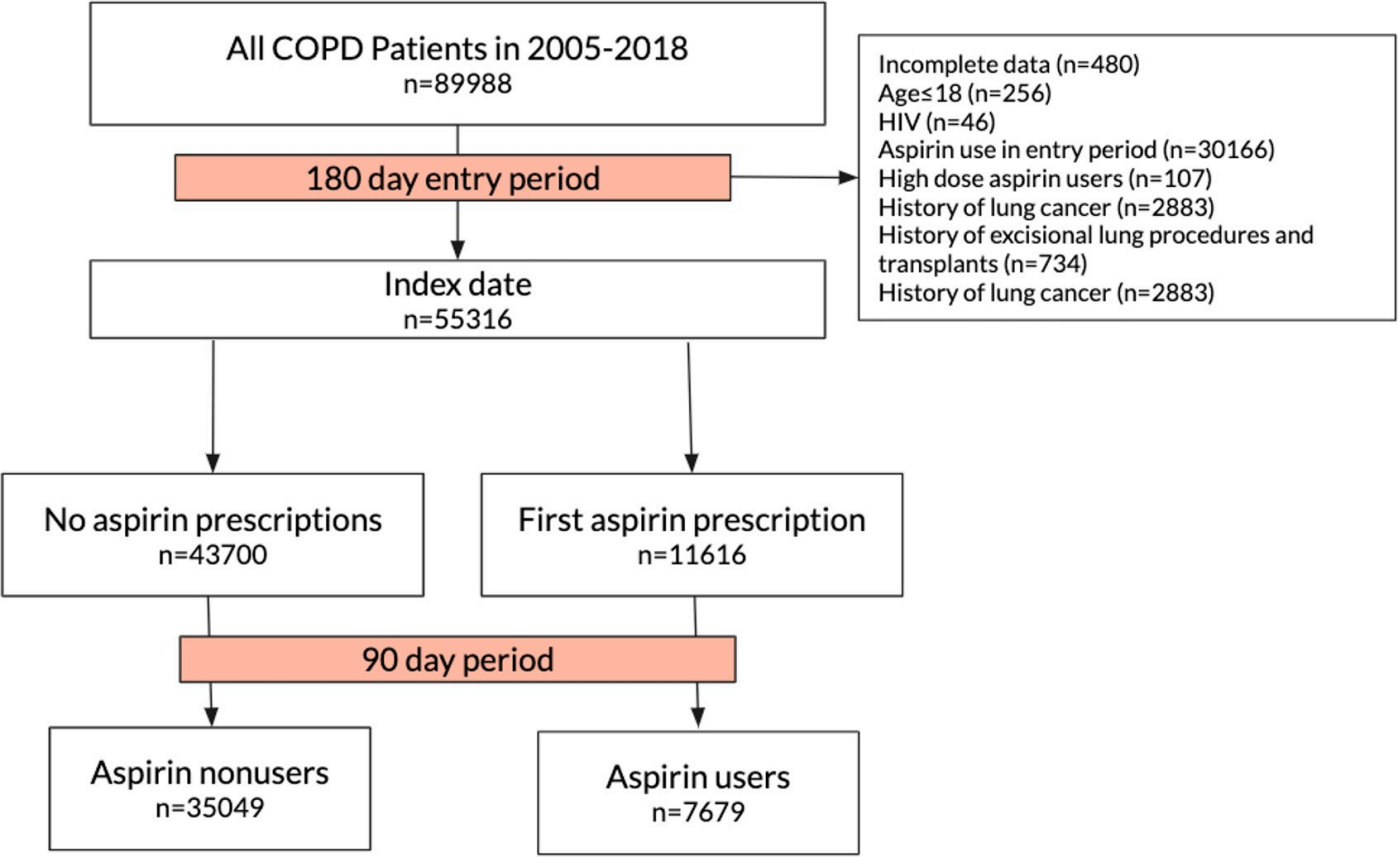
Study cohort selection flow diagram. Patients are excluded during the 90-day period if there are mortalities, diagnoses of lung cancer, excisional lung surgeries, or antiplatelets use other than aspirin. COPD, chronic obstructive pulmonary disease.

Among a total of 89,988 patients with COPD diagnosed between 2005 and 2018, 42,728 patients were eligible, including 35,049 aspirin nonusers and 7,679 aspirin users (Fig 1). The baseline characteristics of the entire cohort are shown in Table 1. The mean age of the cohort was 75.7 years, and majority were men. The propensity score model yielded a C-statistic of 0.703. Upon adjustment by IPTW, patient characteristics were well balanced (Table 1, S2 and S3 Tables). Among aspirin users, 1,330 (17.3%) had vascular diseases and/or coronary artery disease, and 3,198 (41.6%) had at least 1 cardiovascular risk factor (i.e., diabetes, obesity, hypertension, or dyslipidemia requiring lipid-lowering agent).

**Table 1:** Baseline characteristics of the study cohort, including their standardized mean difference before and after inverse propensity of treatment weighting.

Characteristic (%)	All (*N* = 42,728)	Aspirin nonusers (*N* = 35,049)	Aspirin users (*N* = 7,679)	Standardized mean difference before IPTW	Standardized mean difference after IPTW
Male sex—no. (%)	34,757 (81.3)	28,715 (81.9)	6,042 (78.7)	0.082	0.008
Age at index date—mean years ± SD	75.7 ± 11.5	75.1 ± 11.9	78.6 ± 9.0	0.329	0.086
Intravenous drug use—no. (%)	77 (0.2)	66 (0.2)	11 (0.1)	0.011	0.008
Nonsmoking etiologies—no. (%)	6,428 (15.0)	5,323 (15.2)	1,105 (14.4)	0.011	0.003
Inpatient visits in past year—mean no. ± SD	2.07 ± 3.16	1.92 ± 3.18	2.76 ± 2.97	0.271	0.054
Diabetes—no. (%)	3,638 (8.5)	2,670 (7.6)	968 (12.6)	0.166	0.023
Obesity—no. (%)	130 (0.3)	92 (0.3)	38 (0.5)	0.038	0.008
Hypertension—no. (%)	9,408 (22.0)	7,000 (20.0)	2,408 (31.4)	0.263	0.027
Cerebrovascular disease—no. (%)	2,210 (5.2)	1,332 (3.8)	878 (11.4)	0.291	0.015
Peripheral vascular disease—no. (%)	609 (1.4)	378 (1.1)	231 (3.0)	0.137	0.014
Congestive heart failure—no. (%)	3,783 (8.9)	2,560 (7.3)	1,223 (15.9)	0.272	0.024
Coronary artery disease—no. (%)	550 (1.3)	214 (0.6)	336 (4.4)	0.243	0.031
Arrhythmia—no. (%)	3,014 (7.1)	1,724 (4.9)	1,290 (16.8)	0.389	0.006
Gastrointestinal bleeding—no. (%)	2,614 (6.1)	2,123 (6.1)	491 (6.4)	0.014	0.029
Nongastrointestinal bleeding—no. (%)	3,085 (7.2)	2,543 (7.3)	542 (7.1)	0.008	0.010
Liver cirrhosis—no. (%)	494 (1.2)	405 (1.2)	89 (1.2)	0.001	0.002
Coagulation defects—no. (%)	45 (0.1)	36 (0.1)	9 (0.1)	0.004	0.002
Inhaled steroid use—no. (%)	24,082 (56.4)	19,493 (55.6)	4,589 (59.8)	0.084	0.015
Bronchodilator use					
Beta agonist—no. (%)	4,380 (10.3)	3,625 (10.3)	757 (9.9)	0.016	0.007
Antimuscarinics—no. (%)	5,031 (11.8)	4,159 (11.9)	872 (11.4)	0.016	0.004
Both beta agonists and antimuscarinics—no. (%)	20,373 (47.7)	19,365 (45.7)	3,988 (51.9)	0.106	0.022
Methylxanthines—no. (%)	11,191 (26.2)	8,976 (25.6)	2,215 (28.8)	0.073	0.007
Others respiratory medications—no. (%)	994 (2.3)	847 (2.4)	147 (1.9)	0.035	0.003
Antidepressant use					
SSRI—no. (%)	1,059 (2.5)	854 (2.4)	205 (2.7)	0.015	0.012
SNRI—no. (%)	108 (0.3)	86 (0.2)	22 (0.3)	0.008	0.003
Tricyclic antidepressants—no. (%)	516 (1.2)	391 (1.1)	125 (1.6)	0.044	0.002
Others—no. (%)	605 (1.4)	479 (1.4)	126 (1.6)	0.023	0.001
Insulin use—no. (%)	1,469 (3.4)	1,081 (3.1)	388 (5.1)	0.100	0.016
Antidiabetic use					
Metformin—no. (%)	3,136 (7.3)	2,354 (6.7)	782 (10.2)	0.125	0.018
Incretin related—no. (%)	236 (0.6)	196 (0.6)	40 (0.5)	0.005	0.011
Sulfonylureas—no. (%)	3,125 (7.3)	2,313 (6.6)	812 (10.6)	0.142	0.016
SGLT2 inhibitors—no. (%)	12 (0.0)	10 (0.0)	2 (0.0)	0.002	0.007
Thiazolidinediones—no. (%)	31 (0.1)	22 (0.1)	9 (0.1)	0.018	0.001
Others—no. (%)	52 (0.1)	41 (0.1)	11 (0.1)	0.007	0.005
NSAID use	1,832 (4.3)	1,419 (4.0)	413 (5.4)	0.063	0.005
Antihypertensive use					
Alpha-blockers—no. (%)	7,673 (18.0)	5,976 (17.1)	1,697 (22.1)	0.128	0.030
Beta-blockers—no. (%)	2,519 (5.9)	1,922 (5.5)	597 (7.8)	0.092	0.041
Calcium channel blockers—no. (%)	15,039 (35.2)	11,560 (33.0)	3,479 (45.3)	0.255	0.036
Diuretics—no. (%)	8,426 (19.7)	6,306 (18.0)	2,120 (27.6)	0.231	0.036
ACE inhibitors—no. (%)	6,952 (16.3)	5,122 (14.6)	1,830 (23.8)	0.236	0.021
Angiotensin receptor blockers—no. (%)	1,418 (3.3)	1,097 (3.1)	321 (4.2)	0.056	0.027
Others—no. (%)	2,183 (5.1)	1,601 (4.6)	582 (7.6)	0.126	0.012
Lipid-lowering drug					
Statins–no. (%)	3,752 (8.8)	2,864 (8.2)	888 (11.6)	0.114	0.037
Fibrates–no. (%)	271 (0.6)	182 (0.5)	89 (1.2)	0.070	0.002
Others—no. (%)	25 (0.1)	19 (0.1)	6 (0.1)	0.009	0.004

A variable is considered balanced between users and nonusers where the standardized mean difference is ≤0.1.

ACE, angiotensin converting enzyme; IPTW, inverse probability of treatment weighting; NSAID, nonsteroidal anti-inflammatory drug; SD, standard deviation; SGLT2, sodium–glucose transport protein 2; SNRI, serotonin–noradrenaline reuptake inhibitor; SSRI, selective serotonin reuptake inhibitor.

### Lung carcinoma

**Fig 2:**
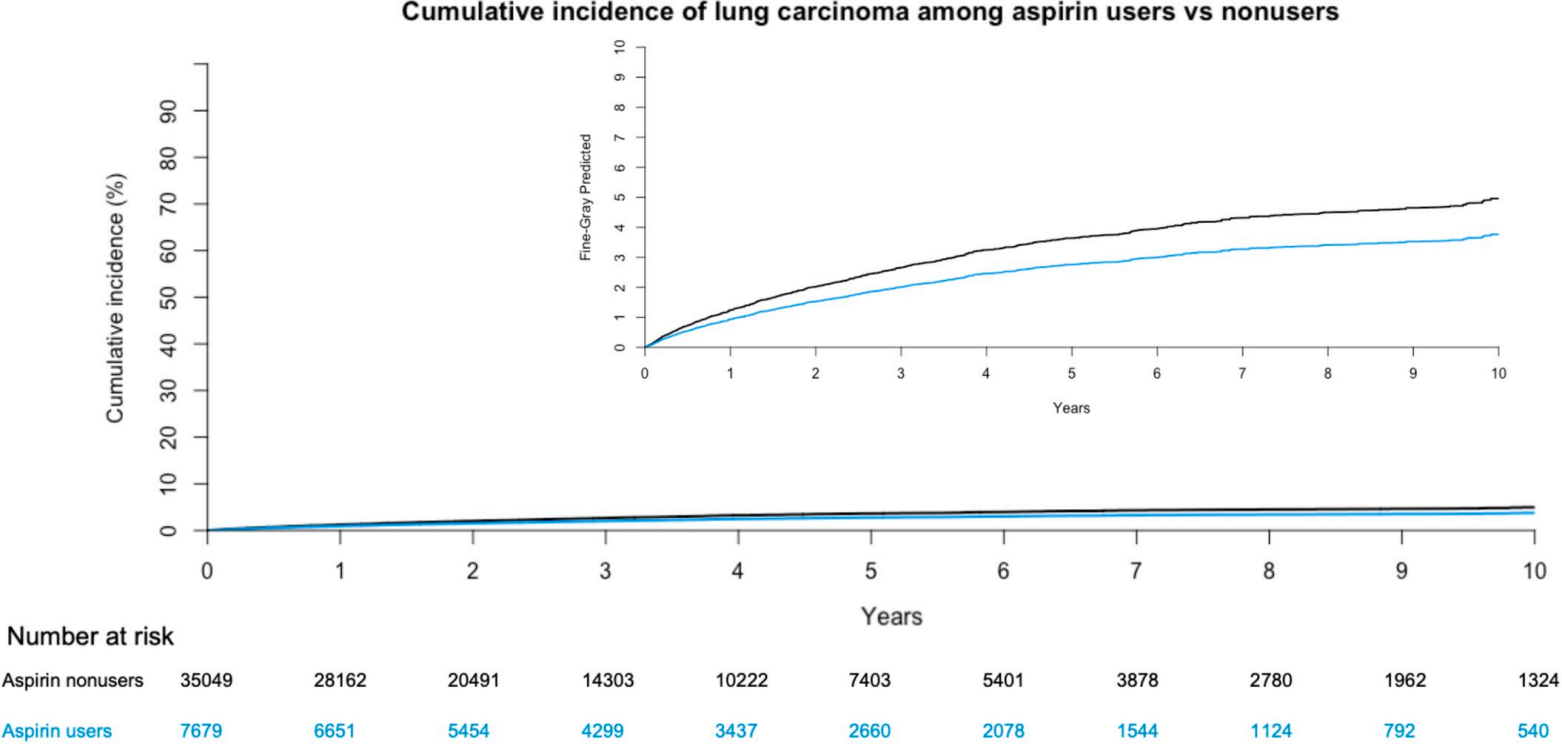
Cumulative incidence of lung carcinoma in aspirin and nonusers. Results were weighted by inverse probability of treatment and multivariate adjusted for age at index date, sex, comorbidities (diabetes, obesity, hypertension, cerebrovascular diseases, peripheral vascular diseases, congestive heart failure, coronary heart disease, arrhythmias, gastrointestinal bleeding and nongastrointestinal bleeding, cirrhosis, and coagulation defects), and drug history (use of antihypertensives, insulin, antidiabetics, beta-blockers, bronchodilators, insulin, NSAIDs, lipid-regulating drugs, and inhaled steroids) as well as factors relating to their socioeconomic status (alcoholism, nonsmoking etiologies of COPD, intravenous drug use, and number of inpatient hospital visits in the year prior to index), accounting for competing risks. The inset shows the same diagram with an expanded y-axis. Day 0 on the x-axis represents 90 days after index date. COPD, chronic obstructive pulmonary disease; NSAID, nonsteroidal anti-inflammatory drug.

During a median follow-up of 2.6 years (interquartile range [IQR]: 1.4 to 4.8), with a total of 148,683 person-years, 1,779 patients (4.2%) were diagnosed with lung carcinoma. The median age of diagnosis of lung carcinoma was 78.0 years (IQR: 71.8 to 83.4 years), and the median time to diagnosis of lung carcinoma beginning from index date was 1.74 years (IQR: 0.79 to 3.25 years). Among aspirin users, the 3-year and 5-year cumulative incidence of lung carcinoma was 2.0% and 2.8%, respectively; while among aspirin nonusers, it was 2.7% and 3.6%, respectively (Fig 2). Aspirin users had a 25% lower risk of lung carcinoma than nonusers after multivariable adjustment (Table 2, SHR = 0.75, 95% confidence interval [CI] 0.65 to 0.87, *p* < 0.001). At the third year and the fifth year, this translated to an NNT of 143 and 125, respectively.

**Table 2:** Association between aspirin use and lung carcinoma.

	Number of patients	Number of outcomes	Person-years of follow-up	Unadjusted SHR (95% CI)	Adjusted SHR (95% CI)	*p*-Value on adjustment
Lung carcinoma						
Aspirin nonusers	35,049	1,526	115,442.7	1.00 (Ref)	1.00 (Ref)	
Aspirin users	7,679	253	33,240.9	0.53 (0.30 to 0.95)	0.75 (0.65 to 0.87)	<0.001
Nonsmall cell lung carcinoma						
Aspirin nonusers	35,049	615	115,442.7	1.00 (Ref)	1.00 (Ref)	
Aspirin users	7,679	101	33,240.9	0.81 (0.65 to 1.01)	0.84 (0.67 to 1.06)	0.136
Small cell lung carcinoma						
Aspirin nonusers	35,049	102	115,442.7	1.00 (Ref)	1.00 (Ref)	
Aspirin users	7,679	14	33,240.9	0.50 (0.26 to 0.95)	0.53 (0.30 to 0.95)	0.030
Lung carcinoma–associated mortality						
Aspirin nonusers	35,049	1,450	116,763.1	1.00 (Ref)	1.00 (Ref)	
Aspirin users	7,679	244	33,488.8	0.73 (0.63 to 0.85)	0.74 (0.64 to 0.86)	<0.001

Variables included in the adjusted model include age at index date, sex, comorbidities (diabetes, obesity, hypertension, cerebrovascular diseases, peripheral vascular diseases, congestive heart failure, coronary heart disease, arrhythmias, gastrointestinal bleeding and nongastrointestinal bleeding, cirrhosis, and coagulation defects), and drug history (use of antihypertensives, insulin, antidiabetics, beta-blockers, bronchodilators, insulin, NSAIDs, lipid-regulating drugs, and inhaled steroids) as well as factors relating to their socioeconomic status (alcoholism, nonsmoking etiologies of COPD, intravenous drug use, and number of inpatient hospital visits in the year prior to index).

CI, confidence interval; COPD, chronic obstructive pulmonary disease; NSAID, nonsteroidal anti-inflammatory drug; SHR, subdistribution hazard ratio.

A total of 26,186 mortalities occurred during follow-up, among which 1,694 (6.0%) were attributed specifically to lung carcinoma. Upon accounting for nonlung carcinoma causes of mortality and excisional lung procedures as competing risks, aspirin use was associated with a 26% decrease in lung carcinoma–related mortality compared to nonusers (SHR = 0.74, 95% CI 0.64 to 0.86, *p* < 0.001).

Histological classifications of lung carcinoma were retrievable and reported in 832 cases (Table 3). This included 116 episodes of small cell lung carcinoma and 716 episodes of nonsmall cell lung carcinoma. The use of aspirin was significantly associated with lower chance for small cell carcinoma compared to nonusers (SHR = 0.53, 95% CI 0.30 to 0.95, *p* = 0.03), whereas there was no statistically significant difference in the hazard for nonsmall cell carcinoma between aspirin users and nonsuers (SHR = 0.84, 95% CI 0.67 to 1.06, *p* = 0.14).

**Table 3:** Details regarding histological types of lung carcinoma in 832 patients.

Histological description	Count	Aspirin users	Aspirin nonusers
Nonsmall cell carcinoma	716	101	615
Adenocarcinoma	298	39	259
Squamous cell carcinoma	248	38	210
Large cell carcinoma	1	0	1
Adenosquamous carcinoma	1	0	1
Bronchioloalveolar carcinoma	3	1	2
Spindle cell carcinoma	3	1	2
Mucoepidermoid carcinoma	1	1	0
Acinar adenocarcinoma	1	0	1
Atypical carcinoid tumor	1	1	0
Unspecified nonsmall cell carcinoma	159	20	139
Small cell carcinoma	116	14	102
Total identified	832	115	717

### Sensitivity analysis

Sensitivity analysis for the associations between aspirin and lung carcinoma revealed that when a delayed entry of 4 years was considered, with 10,203 aspirin nonusers and 3,434 aspirin users, aspirin users was associated with a lower risk compared to aspirin nonusers (SHR = 0.75, 95% CI 0.57 to 0.99, *p* = 0.04) (S4 Table). Without propensity matching or considerations of competing risks, a multivariate cox regression yielded a hazard ratio of 0.53 for users compared to nonusers (95% CI 0.30 to 0.95, *p* = 0.03). Without using IPTW, instead including the propensity score as part of the multivariable regression, the association between aspirin use and a lower risk of lung carcinoma compared to nonuse remained (SHR = 0.77, 95% CI 0.67 to 0.88, *p* < 0.001). Results of alternative statistical strategies are available in S5 Table. It is worth noting that a lack of adjustment of comorbidities and drug use had insignificantly contributed, respectively, to a lower and higher point estimate of the association between aspirin and lung carcinoma (S6 Table). Upon excluding 274 diagnoses of COPD in patients aged below 40, aspirin users’ negative association with lung carcinoma was still present compared to nonusers (SHR = 0.75, 95% CI 0.65 to 0.86, *p* < 0.001). When we considered a more stringent criteria for aspirin exposure, instead defining aspirin use as more than 290 DDD of use in the first year since index date, aspirin’s protective association remained compared to aspirin nonusers (SHR = 0.78, 95% CI 0.65 to 0.92, *p* = 0.005). Upon censoring all patients with any history of malignant cancers (*n* = 4,482) or including a history of malignant cancers as an additional variable in performing IPTW and multivariate regression, the SHRs for aspirin users compared to nonusers were 0.75 (95% CI 0.66 to 0.87, *p* < 0.001) and 0.76 (95% CI 0.65 to 0.88, *p* < 0.001), respectively. The E-value was 1.6 for the upper limit of the CI suggesting that for an unmeasured confounder to render the results statistically insignificant, it would need to be very strongly associated with both aspirin use and lung carcinoma (>60% difference in prevalence among users and nonusers and a hazard ratio >1.6/<0.6 on lung carcinoma).

### Subgroup analysis

Results of subgroup analyses were summarized in Table 4. We found that aspirin users, compared to nonusers, were associated with lower incidence of lung cancer in both groups aged >75 or ≤75 years, in men, nondiabetic patients, nonhypertensive patients, in both inhaled steroid users and nonusers, in bronchodilator users of only antimuscarinics and both antimuscarinics and beta agonists, statin nonusers, and nonusers of NSAIDs other than aspirin. However, given the small size of certain subgroups, the results should be interpreted with caution. Tests for interaction were not significant for all of the variables investigated.

**Table 4:** Subgroup analyses of association between aspirin use and lung carcinoma hazard.

Characteristic	Aspirin status	Number of patients	Lung carcinoma cases	Person-years of follow-up	Unadjusted SHR (95% CI)	*p*-Value by univariate Cox regression	Adjusted SHR (95% CI)	*p*-Value by multivariate Cox regression	*p*-Value for interaction
Age									
≤75 years old	Nonusers Users	15,495 2,348	756 97	60,309.1 12,802.9	1.00 (Ref) 0.67 (0.54 to 0.84)	0.0005	1.00 (Ref) 0.68 (0.54 to 0.86)	0.001	
>75 years old	Nonusers Users	19,554 5,331	770 156	55,133.6 20,438.0	1.00 (Ref) 0.80 (0.67 to 0.95)	0.0138	1.00 (Ref) 0.74 (0.62 to 0.88)	0.001	0.378
Sex									
Female	Nonusers Users	6,334 1,638	201 44	20,501.5 7,046.4	1.00 (Ref) 0.83 (0.59 to 1.17)	0.2890	1.00 (Ref) 0.85 (0.60 to 1.21)	0.371	
Male	Nonusers Users	28,715 6,042	1,325 209	94,941.2 26,194.5	1.00 (Ref) 0.72 (0.62 to 0.85)	<0.0001	1.00 (Ref) 0.74 (0.64 to 0.87)	<0.001	0.582
Diabetic									
No	Nonusers Users	32,379 6,711	1,434 226	107,827.2 29,927.3	1.00 (Ref) 0.73 (0.63 to 0.85)	0.0001	1.00 (Ref) 0.75 (0.65 to 0.87)	<0.001	
Yes	Nonusers Users	2,670 968	92 27	7,615.4 4,013.6	1.00 (Ref) 0.89 (0.55 to 1.40)	0.5880	1.00 (Ref) 0.77 (0.49 to 1.21)	0.259	0.757
Hypertensive									
No	Nonusers Users	28,049 5,271	1,288 238	96,369.5 23,812.4	1.00 (Ref) 0.71 (0.60 to 0.84)	<0.0001	1.00 (Ref) 0.73 (0.62 to 0.86)	<0.001	0.429
Yes	Nonusers Users	7,000 2,408	183 70	19,073.2 9,428.6	1.00 (Ref) 0.87 (0.65 to 1.18)	0.3650	1.00 (Ref) 0.82 (0.62 to 1.08)	0.156	
Inhaled steroid use									
No	Nonusers Users	15,556 3,090	771 119	54,445.7 14,768.6	1.00 (Ref) 0.78 (0.64 to 0.96)	0.0164	1.00 (Ref) 0.79 (0.64 to 0.96)	0.021	
Yes	Nonusers Users	19,493 4,589	815 134	60,097.0 18,472.3	1.00 (Ref) 0.70 (0.58 to 0.86)	0.0004	1.00 (Ref) 0.72 (0.60 to 0.96)	0.001	0.964
Bronchodilator use									
Neither	Nonusers Users	10,882 2,062	433 86	42,343.3 10,533.4	1.00 (Ref) 0.90 (0.71 to 1.15)	0.4110	1.00 (Ref) 0.88 (0.68 to 1.14)	0.328	
Beta agonist only	Nonusers Users	3,623 757	145 18	12,833.7 3,587.5	1.00 (Ref) 0.55 (0.34 to 0.91)	0.1990	1.00 (Ref) 0.60 (0.36 to 1.01)	0.056	0.093
Antimuscarinics only	Nonusers Users	4,159 872	214 31	13,037.5 3,733.5	1.00 (Ref) 0.63 (0.42 to 0.95)	0.0265	1.00 (Ref) 0.65 (0.43 to 0.99)	0.045	0.113
Both	Nonusers Users	16,385 4,008	734 118	47,228.0 15,386.5	1.00 (Ref) 0.71 (0.58 to 0.87)	0.0012	1.00 (Ref) 0.71 (0.58 to 0.87)	0.001	0.939
Statin use									
No	Nonusers Users	32,185 6,791	1,446 80	107,250.7 29,564.9	1.00 (Ref) 0.71 (0.61 to 0.82)	<0.0001	1.00 (Ref) 0.73 (0.62 to 0.85)	<0.001	
Yes	Nonusers Users	2,864 888	223 30	8,192.0 3,676.0	1.00 (Ref) 1.23 (0.79 to 1.9)	0.3630	1.00 (Ref) 1.07 (0.68 to 1.70)	0.777	0.054
Nonaspirin NSAID									
No	Nonusers Users	33,630 7,266	1,466 240	110,440.0 31,246.3	1.00 (Ref) 0.74 (0.64 to 0.85)	<0.0001	1.00 (Ref) 0.87 (0.75 to 0.65)	<0.001	
Yes	Nonusers Users	1,419 4,413	60 13	5,002.7 1,994.6	1.00 (Ref) 0.74 (0.40 to 1.36)	0.3290	1.00 (Ref) 0.87 (0.50 to 1.53)	0.6341	0.9392

Variables included in the adjusted model include age at index date, sex, comorbidities (diabetes, obesity, hypertension, cerebrovascular diseases, peripheral vascular diseases, congestive heart failure, coronary heart disease, arrhythmias, gastrointestinal bleeding and nongastrointestinal bleeding, cirrhosis, and coagulation defects), and drug history (use of antihypertensives, insulin, antidiabetics, beta-blockers, bronchodilators, insulin, NSAIDs, lipid-regulating drugs, and inhaled steroids) as well as factors relating to their socioeconomic status (alcoholism, nonsmoking etiologies of COPD, intravenous drug use, and number of inpatient hospital visits in the year prior to index).

CI, confidence interval; COPD, chronic obstructive pulmonary disease; NSAID, nonsteroidal anti-inflammatory drug; SHR, subdistribution hazard ratio.

### Bleeding

A total of 12,581 diagnoses of bleeding were recorded between index date and patient mortality among 7,194 patients (Table 5). Poisson regression yielded a count ratio of 1.33 for aspirin users compared to nonusers upon adjustment for age, sex, comorbidities, concurrent drug uses, and proxy factors for socioeconomic status as stated in the Methods section (95% CI 1.30 to 1.36, *p* < 0.001). Analyzing only the survival function of time to the first bleeding episode, the SHR was 1.78 for aspirin users compared to nonusers on multivariate adjustment (95% CI 1.68 to 1.89, *p* = 0.001). Compared to nonusers, aspirin use was not associated with UGIB in particular on multivariate adjustment (SHR = 1.19, 95% CI 0.94 to 1.53, *p* = 0.16), with the 5-year incidence being 0.7% and 0.8%, respectively. However, aspirin users were strongly associated with a higher risk of hemoptysis compared to nonusers on multivariate adjustment (SHR = 1.96, 95% CI 1.73 to 2.23, *p* < 0.001). The risk ratios of aspirin on overall bleeding, UGIB, and hemoptysis were slightly attenuated when aspirin users are censored upon initiation of other antiplatelets while remaining statistically significant (S7 Table). Unadjusted results can be found in Table 5 and S7 Table. Since aspirin was likely to be discontinued upon an episode of bleeding, we performed a sensitivity analysis of the association between aspirin and lung carcinoma taking into account bleeding as an additional competing risk, in which case aspirin use was associated with 30% lower lung carcinoma incidence compared to nonuse (SHR 0.70, 95% CI 0.60 to 0.82, *p* < 0.001).

**Table 5:** Counts of bleeding episodes among aspirin users and nonusers over lifetime.

Patient cohort	Number of bleeding diagnoses	Person-years follow-up	Diagnoses per thousand person-years	Relative risk (unadjusted)	Relative risk (adjusted)
Aspirin nonusers	8,689	116,763.14	74.4	1.00 (Ref)	1.00 (Ref)
Aspirin users	3,892	33,488.79	116.2	0.46 (0.44 to 0.49)	1.33 (1.30 to 1.36)

Variables included in the adjusted model include age at index date, sex, comorbidities (diabetes, obesity, hypertension, cerebrovascular diseases, peripheral vascular diseases, congestive heart failure, coronary heart disease, arrhythmias, gastrointestinal bleeding and nongastrointestinal bleeding, cirrhosis, and coagulation defects), and drug history (use of antihypertensives, insulin, antidiabetics, beta-blockers, bronchodilators, insulin, NSAIDs, lipid-regulating drugs, and inhaled steroids) as well as factors relating to their socioeconomic status (alcoholism, nonsmoking etiologies of COPD, intravenous drug use, and number of inpatient hospital visits in the year prior to index).

COPD, chronic obstructive pulmonary disease; NSAID, nonsteroidal anti-inflammatory drug.

## Discussion

In this territory-wide cohort study of more than 42,000 patients with COPD, we demonstrated that aspirin use was associated with a 25% decrease in the risk of developing lung carcinoma and 26% decrease in lung carcinoma–related mortality. Subgroup analysis further showed that the risk reduction of aspirin users were consistent in those who were >75 and ≤75 years of age, men, nondiabetic, nonhypertensive, statin nonusers, and nonusers of NSAID other than aspirin. Nonetheless, the benefits were accompanied by a substantially higher incidence of hemoptysis.

The current study provides evidence on the relationship between aspirin and lung carcinoma in patients with COPD through thorough consideration of potential sources of confounding and biases achieved by propensity score analytics. Numerous reports have suggested that aspirin may reduce carcinogenesis in various groups of patients. In a nationwide study, regular use of aspirin has been shown to reduce hepatocellular carcinoma [22]. Similarly, large population-based studies have also demonstrated that the use of low-dose aspirin may reduce prostate cancer [10], epithelial ovarian cancer [8], and colorectal cancer [9]. Nonetheless, the role of aspirin in the prevention of lung carcinoma is controversial. A large cohort study involving health screening participants has demonstrated that the use of low-dose aspirin for more than 5 years decreases the risk of lung carcinoma by 4%, particularly among elderly and nondiabetic patients [14]. Similarly, lung carcinoma mortality was dose dependently reduced with aspirin use in another population-based nationwide cohort [33]. A pooled analysis of randomized trials for primary and secondary prevention of vascular events revealed that daily aspirin use reduced lung cancer–related mortality compared to a control group [34]. By contrast, 2 prospective cohort studies [35,36] and recent meta-analyses failed to demonstrate an association between aspirin and development of lung carcinoma [16]. These nonrandomized studies without consideration for some indications of aspirin, however, may be susceptible to biases in treatment selection and were also limited in their consideration of confounders including concurrent drug uses.

In addition to supporting previous studies, we found that aspirin’s chemoprotective association is present even in a relatively short follow-up period in patients with COPD, compared to previous studies where the protective association of aspirin is only statistically significant after prolonged follow-up periods (>5 years) in the general population [14]. The shorter follow-up duration before a statistically significant chemoprotective association is observed, as in the present study, could be partially explained by the high baseline hazard to lung carcinoma among patients with COPD—smokers with COPD had a 5-fold risk of lung carcinoma compared to smokers with normal lung functions, implying that the association could be uncovered despite a smaller cohort [3]. While some may be inclined to think of this as the sole reason, however, this study estimates a CI for the SHR associated with aspirin use, which is significantly lower than previous estimates, despite similar analytical approaches. This suggests that the relative chemoprotective association is considerably stronger among patients with COPD than in the general population. This, in clinical settings, would imply a lower NNT. The clinical implications of an association between lung carcinoma incidence and aspirin use is especially relevant among patients with COPD, who are not only more susceptible to lung carcinoma, but in whom lung carcinoma tend also to be more aggressive [37].

Mechanisms of aspirin’s protective associations in patients with COPD are uncertain but have been postulated to be related to both anti-inflammatory and antiplatelet properties of aspirin [12]. Aspirin, although considered a nonspecific cyclooxygenase inhibitor, is more specific toward COX-1 at low doses [38]. Clinical studies have shown COX-1 activity as the primary driver of changes in prostanoids of exhaled breath condensates in COPD [13]. Prostanoids including thromboxane A and prostaglandin E2, which are inhibited by low-dose aspirin, may contribute to crosstalk among platelets and cells of the tumor environment [39]. The inhibition of platelets by aspirin may also retard tumor progression and lower the risk of epithelial-to-mesenchymal transition, a process that promotes malignant transformation in the respiratory epithelium [40–42]. Moreover, contents of platelet α-granules can also induce COX-2 expression [39]. DNA sequence variations in the COX-2 gene as well as in enzymes relevant in the metabolism of arachidonic acid into eicosanoids is associated with differing risks of lung carcinoma development of diverse histological subtypes [43–46]. Indeed, preclinical evidences suggest that the level of COX-2 expression is increased in patients with COPD, thus perhaps potentiating the chemoprotective effects of aspirin as compared to the general population [47]. Additionally, histological series have shown that both isoforms of cyclooxygenase are expressed in a subset of lung tumors [48]. Finally, aspirin has also been demonstrated to result in a reduction in acute exacerbations, mortality, and progression in COPD, which is in itself a risk factor for lung carcinoma [4,49,50]. Given the multiple pathways via which aspirin’s chemoprotective effects can be mediated in COPD, it is biologically plausible that any chemoprotective effect of aspirin is stronger among patients with COPD compared to the general population.

Bleeding risk, in particular UGIB, is one of the major complications of chronic aspirin use. Large population-based studies evaluating the chemoprotective associations of aspirin have shown that UGIB risk was not increased in aspirin users [22,51,52]. Similarly, our result corroborates with prior findings of a lack of association between aspirin use and a higher risk of UGIB. Nonetheless, our results show that aspirin use was associated with a significantly higher risk of hemoptysis; this has not been reported in other similar studies involving patients with COPD to the best of our knowledge [4,5].

Strengths of the present study included the use of a territory-wide, well-validated electronic healthcare database with records of all diagnoses, hospitalizations, and details of drug dispenses, allowing the collection of relevant information required to preclude common biases in conventional observational studies such as selection and recall biases. The adjustment of potential chemoprotective agents like statins [53] and inhaled corticosteroids [54] further increases the validity of the present result as the apparent protective effect of aspirin in previous studies could be linked to the common prescription of aspirin and statin in cardiovascular diseases. The application of IPTW to an unselected population with COPD with detailed clinical and medication history provided compelling evidence regarding potential benefits of aspirin in preventing lung carcinoma. Sensitivity analysis with different propensity score models and competing risk analyses to address potential biases further validated our study results. The possibility of reverse causation is minimal considering the similarly statistically significant result for a cohort of patients studied beginning from 4 years after aspirin use.

There were several limitations in the present study. First of all, the staging of lung carcinoma was not available. Risk factors such as family history of lung carcinoma were also not available. It is commonly recognized, however, that variables such as family history has no apparent effect on drug prescription, thus conferring little confounding effect in drug–cancer association studies [55]. Furthermore, the details of smoking, including pack-years of smoking, smoking duration, and smoking status at index date, were not recorded in the electronic system. However, as demonstrated in prior studies, not only were the percentages of smokers similar among aspirin users and nonusers, but prior studies have also shown that aspirin’s protective association with lung carcinoma was irrespective of smoking history [14]. In addition, due to the lack of histological evaluation in some patients with lung carcinoma, histological diagnoses were only available in 47% of patients. While this does not affect the validity of the main analyses, it limited our insight into associations between aspirin and the risk of specific cancer types where there are insufficient cases. Moreover, the observational nature of the study implied that the association may be partially mediated by the healthy user effect. While this source of error cannot be eliminated, the use of propensity score analytics may be able to capture proxy variables that likely reflect health-seeking tendencies and ability of patients to access healthcare [56]. In our study, the propensity score has taken into account variables including number of inpatient visits in the prior year and the medications to which the patients were adherent to prior to index date. The balance of these variables between aspirin users and nonusers upon IPTW minimizes the effect of the healthy user effect. In addition, it is possible that residual confounders remained despite utilizing propensity score analytics. Such confounders would, however, need to be very significant in order to invalidate results of this study. Finally, it may be the case that the result findings may not be applicable in other regions as the majority of our cohort is Chinese.

Lung carcinoma is the most common cause of cancer globally. While emerging techniques such as targeted pharmacotherapies are progressively improving the prognostic outlook among patients with lung carcinoma, investigations into prophylactic management modalities among high-risk cohorts will likely yield cost-effective strategies against lung carcinoma and must therefore not be overlooked. While the current retrospective study should not to be considered sufficient evidence for the inclusion of aspirin in the management strategy for COPD, the protective association between aspirin and lung carcinoma merits elucidation by future randomized studies to thoroughly study its benefits and associated adverse effects, to allow for rigorous cost-effective analyses, and to improve patient autonomy in clinical decision-making.

In this large cohort of patients with COPD, we have demonstrated that aspirin use was associated with a reduced risk of developing lung carcinoma and lung carcinoma–related mortality. Although UGIB was similar between users and nonusers, aspirin users had a higher incidence of hemoptysis than nonaspirin users.

## Supporting information

S1 Table Definitions of covariates. (DOCX)

S2 Table Characteristics of the IPTW-treated cohort. IPTW, inverse probability of treatment weighting.
(DOCX)

S3 Table Distribution of propensity score according to treatment group. (DOCX)

S4 Table Aspirin use and risk of incident lung carcinoma with follow-up beginning from fourth year. (DOCX)

S5 Table Aspirin use and risk of incident lung carcinoma in IPTW and alternative analysis strategies. IPTW, inverse probability of treatment weighting.
(DOCX)

S6 Table Aspirin use and risk of incident lung carcinoma without adjusting for selected covariates. (DOCX)

S7 Table Risk of gastrointestinal bleeding events according to aspirin use. (DOCX)

S1 File IRB application form. IRB, institutional review board.
(PDF)

S2 File RECORD-PE Checklist. RECORD-PE, REporting of studies Conducted using Observational Routinely collected Data for Pharmacoepidemiology.
(DOCX)

## Abbreviations

CDARS: Clinical Data Analysis Reporting System
CI: confidence interval
COPD: chronic obstructive pulmonary disease
DDD: defined daily dose
IPTW: inverse probability of treatment weighting
IQR: interquartile range
IRB: institutional review board
NNT: number needed to treat
NSAID: nonsteroidal anti-inflammatory drug
RECORD: REporting of studies Conducted using Observational Routinely collected Data
SHR: subdistribution hazard ratio
UGIB: upper gastrointestinal bleeding
