# Supplementary material for: Low-dose aspirin and incidence of lung carcinoma in patients with chronic obstructive pulmonary disease in Hong Kong: A cohort study
Source: PLoS Med. 2022 Jan 13;19(1):e1003880. doi: 10.1371/journal.pmed.1003880 (PMC8757901; doi:10.1371/journal.pmed.1003880)
Supplement: S1 Table — (DOCX) [file pmed.1003880.s001.docx]

**S1 Table.** Definitions of covariates

| Entity | Definition |
| --- | --- |
| Diabetes | ICD-9: 250, 648.0 |
| Obesity | ICD-9: 278 |
| Hypertension | ICD-9: 401-405 |
| Cerebrovascular disease | ICD-9: 430-438 |
| Peripheral vascular disease | ICD-9: 440-444, 447 |
| Congestive heart failure | ICD-9: 402, 404, 425, 428 |
| Coronary artery disease | ICD-9: 410-412, 413 |
| Arrhythmia | ICD-9: 427 |
| Gastrointestinal bleeding | ICD-9: 456.0, 531-533, 578 |
| Non gastrointestinal bleeding | ICD-9: 423.0, 569.3, 786.3, 599.7, 280, 286.5 |
| Liver cirrhosis | ICD-9: 571 |
| Coagulation defects | ICD-9: 286 |
| Intravenous drug use | ICD-9: 305.9 |
| Non-smoking aetiologies | ICD-9: 500-508, 493-494 |
| Chronic obstructive pulmonary disease | ICD-9: 491, 492, 496 |
| Human immunodeficiency virus infection | ICD-9: 42, V08 |
| Lung carcinoma | ICD-9: 162 |
| Other malignancies | ICD-9: 140-209 |
| Excisional lung procedures and transplants | ICD-9-CM: 32.3-32.5, 33.5-33.6 |
