# Supplementary material for: Low-dose aspirin and incidence of lung carcinoma in patients with chronic obstructive pulmonary disease in Hong Kong: A cohort study
Source: PLoS Med. 2022 Jan 13;19(1):e1003880. doi: 10.1371/journal.pmed.1003880 (PMC8757901; doi:10.1371/journal.pmed.1003880)
Supplement: S2 Table — IPTW, inverse probability of treatment weighting. (DOCX) [file pmed.1003880.s002.docx]

**S2 Table.** Characteristics of the Inverse Probability of Treatment Weighting treated cohort

| Characteristic (%) | Aspirin nonusers  (N=43,322.2) | Aspirin users  (N=41,008.4) |
| --- | --- | --- |
| Male sex – number (%) | 35,228.8 (81.3) | 33,215.1 (81.0) |
| Age at index date – years ± SD | 75.6 ± 11.8 | 76.71 ± 9.61 |
| Intravenous drug use – no. (%) | 75.7 (0.2) | 58.5 (0.1) |
| Non-smoking aetiologies – no. (%) | 6,487.5 (15.0) | 6,179.7 (15.1) |
| In patient visits in past year – no. ± SD | 2.7 ± 9.8 | 2.33 ± 2.52 |
| Diabetes – no. (%) | 3,768.5 (8.7) | 3,835.6 (9.4) |
| Obesity – no. (%) | 136.4 (0.3) | 147.6 (0.4) |
| Hypertension – no. (%) | 9,901.7 (22.9) | 9,835.1 (24.0) |
| Cerebrovascular disease – no. (%) | 2,526.6 (5.8) | 2,533.7 (6.2) |
| Peripheral vascular disease – no. (%) | 682.4 (1.6) | 720.5 (1.8) |
| Congestive heart failure – no. (%) | 4,276.2 (9.9) | 4,347.8 (10.6) |
| Coronary artery disease – no. (%) | 820.9 (1.9) | 612.5 (1.5) |
| Arrhythmia – no. (%) | 3,443.3 (7.9) | 3,331.4 (8.1) |
| Gastrointestinal bleeding – no. (%) | 2,670.9 (6.2) | 2,822.5 (6.9) |
| Non gastrointestinal bleeding – no. (%) | 3,268.1 (7.5) | 3,204.2 (7.8) |
| Liver cirrhosis – no. (%) | 507.8 (1.2) | 47.5 (0.1) |
| Coagulation defects – no. (%) | 52.8 (0.1) | 47.5 (0.1) |
| Inhaled steroid use – no. (%) | 24,364.2 (56.2) | 23,375.7 (57.0) |
| Bronchodilator use |  |  |
| Beta agonist – no. (%) | 4,423.3 (10.2) | 4,271.0 (10.4) |
| Antimuscarinics – no. (%) | 5,087.5 (11.7) | 4,862.1 (11.9) |
| Both beta agonists and  antimuscarinics – no. (%) | 20,660.1 (47.7) | 20,005.0 (48.8) |
| Methylxanthines – no. (%) | 11,265.7 (26.0) | 10,783.9 (26.3) |
| Others respiratory medications – no. (%) | 996.8 (2.3) | 926.8 (2.3) |
| Antidepressant use |  |  |
| SSRI – no. (%) | 1,094.6 (2.5) | 1,111.5 (2.7) |
| SNRI – no. (%) | 111.0 (0.3) | 110.7 (0.3) |
| Tricyclic antidepressants– no. (%) | 519.0 (1.2) | 498.7 (1.2) |
| Others – no. (%) | 614.6 (1.4) | 587.6 (1.4) |
| Insulin use – no. (%) | 1,536.7 (3.5) | 1,575.0 (3.8) |
| Antidiabetic use |  |  |
| Metformin – no. (%) | 3,174.6 (7.3) | 3,198.2 (7.8) |
| Incretin related – no. (%) | 241.1 (0.6) | 262.7 (0.6) |
| Sulfonylureas – no. (%) | 3,195.8 (7.4) | 3,195.0 (7.8) |
| SGLT2 inhibitors – no. (%) | 12.2 (0.0) | 17.3 (0.0) |
| Thiazolidinediones – no. (%) | 31.3 (0.1) | 28.5 (0.1) |
| Others – no. (%) | 53.6 (0.1) | 57.5 (0.1) |
| NSAID use | 1,864.2 (4.3) | 1,805.3 (4.4) |
| Antihypertensive use |  |  |
| Alpha blockers – no. (%) | 7,910.3 (18.3) | 7,962.6 (19.4) |
| Beta blockers – no. (%) | 2,660.1 (6.1) | 2,940.1 (7.2) |
| Calcium channel blockers – no. (%) | 15,537.5 (35.9) | 15,412.0 (37.6) |
| Diuretics – no. (%) | 8,996.2 (20.8) | 9,125.3 (22.3) |
| ACE inhibitors – no. (%) | 7,404.8 (17.1) | 7,434.0 (18.1) |
| Angiotensin receptor blockers – no. (%) | 1,500.1 (3.5) | 1,578.6 (3.8) |
| Others – no. (%) | 2,288.5 (5.3) | 2,280.4 (5.6) |
| Lipid lowering drug |  |  |
| Statins–no. (%) | 3,955.2 (9.1) | 4,188.4 (10.2) |
| Fibrates–no. (%) | 284.3 (0.7) | 261.7 (0.6) |
| Others – no. (%) | 25.3 (0.1) | 28.6 (0.1) |

Abbreviations: ACE: angiotensin converting enzyme; NSAID: Nonsteroidal anti-inflammatory drugs; SNRI: Serotonin-noradrenaline reuptake inhibitor; SSRI: Selective serotonin-reuptake inhibitors.
