# Supplementary material for: Low-dose aspirin and incidence of lung carcinoma in patients with chronic obstructive pulmonary disease in Hong Kong: A cohort study
Source: PLoS Med. 2022 Jan 13;19(1):e1003880. doi: 10.1371/journal.pmed.1003880 (PMC8757901; doi:10.1371/journal.pmed.1003880)
Supplement: S3 Table — (DOCX) [file pmed.1003880.s003.docx]

**S3 Table.** Distribution of propensity score according to treatment group

| Decile | Aspirin nonuser | Aspirin user |
| --- | --- | --- |
| 1 | 0.08 (0.01) | 0.09 (0.01) |
| 2 | 0.10 (0.01) | 0.11 (0.00) |
| 3 | 0.11 (0.00) | 0.12 (0.00) |
| 4 | 0.13 (0.00) | 0.14 (0.00) |
| 5 | 0.14 (0.00) | 0.15 (0.00) |
| 6 | 0.16 (0.01) | 0.16 (0.01) |
| 7 | 0.17 (0.01) | 0.18 (0.01) |
| 8 | 0.20 (0.01) | 0.21 (0.01) |
| 9 | 0.27 (0.03) | 0.27 (0.02) |
| 10 | 0.55 (0.18) | 0.43 (0.11) |
