# Supplementary material for: Low-dose aspirin and incidence of lung carcinoma in patients with chronic obstructive pulmonary disease in Hong Kong: A cohort study
Source: PLoS Med. 2022 Jan 13;19(1):e1003880. doi: 10.1371/journal.pmed.1003880 (PMC8757901; doi:10.1371/journal.pmed.1003880)
Supplement: S4 Table — (DOCX) [file pmed.1003880.s004.docx]

**S4 Table.** Aspirin use and risk of Incident lung cancer with follow-up beginning from 4^th^ year

| **Incident CA** | **Aspirin nonuser**  **(N=10,203)** | **Aspirin user**  **(N=3,434)** |
| --- | --- | --- |
| Number of cases | 275 | 80 |
| Unadjusted SHR | 1 | 0.70 (0.38-1.29) |
| Sex and age adjusted SHR | 1 | 0.65 (0.34-1.22) |
| Multivariate adjusted SHR | 1 | 0.75 (0.57-0.99) |

Variables included in the multivariate adjusted model include age at index date, sex, comorbidities (diabetes, obesity, hypertension, cerebrovascular diseases, peripheral vascular diseases, congestive heart failure, coronary heart disease, arrhythmias, gastrointestinal bleeding and non-gastrointestinal bleeding, cirrhosis, coagulation defects), and drug history (use of antihypertensives, insulin, antidiabetics, beta blockers, bronchodilators, insulin, non-steroidal anti-inflammatory drugs, lipid regulating drugs, and inhaled steroids) as well as factors relating to their socioeconomic status (alcoholism, non-smoking aetiologies of chronic obstructive pulmonary disease, and number of inpatient hospital visits in the year prior to index). Abbreviations: SHR, subdistribution hazard
