# Supplementary material for: Low-dose aspirin and incidence of lung carcinoma in patients with chronic obstructive pulmonary disease in Hong Kong: A cohort study
Source: PLoS Med. 2022 Jan 13;19(1):e1003880. doi: 10.1371/journal.pmed.1003880 (PMC8757901; doi:10.1371/journal.pmed.1003880)
Supplement: S5 Table — IPTW, inverse probability of treatment weighting. (DOCX) [file pmed.1003880.s005.docx]

**S5 Table.** Aspirin use and risk of Incident lung carcinoma in IPTW and alternative analysis strategies

| **Model** | **Aspirin nonuser** | **Aspirin user** |
| --- | --- | --- |
| **IPTW treated** |  |  |
| Unadjusted SHR | 1 | 0.74 (0.64-0.85) |
| Age and sex adjusted SHR | 1 | 0.75 (0.65-0.86) |
| Multivariable adjusted SHR | 1 | 0.75 (0.65-0.87) |
| Multivariable adjusted SHR censoring users upon initiation of other antiplatelets | 1 | 0.73 (0.63-0.84) |
| **Propensity score regressed** |  |  |
| SHR adjusted only for propensity score | 1 | 0.78 (0.68-0.89) |
| Age, sex, and propensity score adjusted SHR | 1 | 0.78 (0.68-0.89) |
| Multivariable adjusted SHR | 1 | 0.77 (0.67-0.88) |
| Multivariable adjusted SHR censoring users upon initiation of other antiplatelets | 1 | 0.75 (0.65-0.86) |
| **Competing risks without propensity score consideration SHR** | 1 | 0.78 (0.69-0.90) |
| **Conventional multivariable Cox regression HR** | 1 | 0.53 (0.30-0.95) |

Variables included in the adjusted model include age at index date, sex, comorbidities (diabetes, obesity, hypertension, cerebrovascular diseases, peripheral vascular diseases, congestive heart failure, coronary heart disease, arrhythmias, gastrointestinal bleeding and non-gastrointestinal bleeding, cirrhosis, coagulation defects), and drug history (use of antihypertensives, insulin, antidiabetics, beta blockers, bronchodilators, insulin, non-steroidal anti-inflammatory drugs, lipid regulating drugs, and inhaled steroids) as well as factors relating to their socioeconomic status (alcoholism, non-smoking aetiologies of chronic obstructive pulmonary disease, and number of inpatient hospital visits in the year prior to index). Abbreviations: SHR, Subdistribution hazard ratio; IPTW, Inverse probability of treatment weighting; HR, Hazard ratio
