# Supplementary material for: Low-dose aspirin and incidence of lung carcinoma in patients with chronic obstructive pulmonary disease in Hong Kong: A cohort study
Source: PLoS Med. 2022 Jan 13;19(1):e1003880. doi: 10.1371/journal.pmed.1003880 (PMC8757901; doi:10.1371/journal.pmed.1003880)
Supplement: S7 Table — (DOCX) [file pmed.1003880.s007.docx]

**S7 Table.** Risk of gastrointestinal bleeding events according to aspirin use

| **Bleeding event** | **Aspirin nonuser** | **Aspirin user** |
| --- | --- | --- |
| **Any bleeding event** |  |  |
| Number with event | 5,304 | 1,890 |
| Unadjusted SHR | 1 | 1.52 (1.44-1.60) |
| Age and sex adjusted SHR | 1 | 1.52 (1.43-1.60) |
| Multivariable adjusted SHR | 1 | 1.78 (1.68-1.89) |
| Multivariable adjusted SHR censoring users upon initiation of other antiplatelets | 1 | 1.50 (1.42-1.59) |
| **Upper gastrointestinal bleeding as first bleeding event** |  |  |
| Number with event | 355 | 102 |
| Unadjusted SHR | 1 | 1.21 (0.95-1.54) |
| Age and sex adjusted SHR | 1 | 1.21 (0.94-1.55) |
| Multivariable adjusted SHR | 1 | 1.19 (0.94-1.53) |
| Multivariable adjusted SHR censoring users upon initiation of other antiplatelets | 1 | 1.15 (0.90-1.48) |
| **Haemoptysis as first bleeding event** |  |  |
| Number with event | 1,087 | 453 |
| Unadjusted SHR | 1 | 1.90 (1.68-2.14) |
| Age and sex adjusted SHR | 1 | 1.95 (1.73-1.21) |
| Multivariable adjusted SHR | 1 | 1.96 (1.73-2.23) |
| Multivariable adjusted SHR censoring users upon initiation of other antiplatelets | 1 | 1.92 (1.69-2.19) |

Variables included in the multivariate adjusted model include age at index date, sex, comorbidities (diabetes, obesity, hypertension, cerebrovascular diseases, peripheral vascular diseases, congestive heart failure, coronary heart disease, arrhythmias, gastrointestinal bleeding and non-gastrointestinal bleeding, cirrhosis, coagulation defects), and drug history (use of antihypertensives, insulin, antidiabetics, beta blockers, bronchodilators, insulin, non-steroidal anti-inflammatory drugs, lipid regulating drugs, and inhaled steroids) as well as factors relating to their socioeconomic status (alcoholism, non-smoking aetiologies of chronic obstructive pulmonary disease, and number of inpatient hospital visits in the year prior to index). Abbreviations: SHR, subdistribution hazard ratio
