# Supplementary material for: Low-dose aspirin and incidence of lung carcinoma in patients with chronic obstructive pulmonary disease in Hong Kong: A cohort study
Source: PLoS Med. 2022 Jan 13;19(1):e1003880. doi: 10.1371/journal.pmed.1003880 (PMC8757901; doi:10.1371/journal.pmed.1003880)
Supplement: S1 File — IRB, institutional review board. (PDF) [file pmed.1003880.s008.pdf]

Submission Reference No.(For Office Use) HKWC-2020-0877

醫院管理局 HOSPITAL AUTHORITY

Hospital Authority

Clinical Research Ethics Review Application Form

Applying Cluster	HKWC	IRB/ REC Reference No. (For Office Use)	UW 20-810

Instructions to applicant

1. Cluster Research Ethics Committee/Institutional Review Board ("REC/IRB") is dedicated to oversee clinical studies conducted by Hospital Authority ("HA")/University personnel in the Cluster with the aim of protecting the rights, safety and well-being of the human subjects recruited for the studies. The Applicant / Principal Investigator must be designated to take the final responsibility for protecting the rights, safety and well-being of subjects recruited from the Cluster.

2. Please submit the application via online system, enter all information required and upload relevant application dossier files to the required fields.

3. This form is only fully functional with Microsoft Silverlight. This form can only support retrieval of the following attachments - [1] Common Image files, [2] HTML and XML files, [3] Media files, [4] Microsoft Office files (except *.mdb files), [5] PDF files, [6] Text files.

4. This Form does not support certain symbol and text format adjustment. For example, enter text "beta" instead of symbol "β" or copy and paste "β" from another source, and use symbol ^ to indicate "power", e.g. 4x10^3 instead of $4x10^3$.

5. Research protocol, investigator brochures, consent forms, and written materials to subjects must be uniquely identified,for example, by Application Reference Number, document numbers, version numbers and dates.

6. For information on research ethics and methodology, please visit HA Research Ethics Intranet Website: http://cetm.home/ces/re/Home.aspx

7. The handling and storage for data containing personal identity must comply with HA Clinical Data Policy Manual and other prevailing HA policies and if applicable university policies.

8. Selected information will be passed to the HA's and University's Central Register of Clinical Research for the purpose of central record and risk management.

9. Refer to Submission Highlights and submit required number of printed and electronic-copies of signed Application Form and application dossier to the Cluster REC/IRB office of HKWC at Room 901, Administration Block, Queen Mary Hospital, 102 Pokfulam Road, HK.

Reminder

1. Hospital Authority as a Research Institution

The HA is a body corporate established under the Hospital Authority Ordinance (Chapter 113 of the laws of Hong Kong). In addition to the primary responsibilities of establishing, managing, controlling and developing the public hospital system in Hong Kong and advising the Hong Kong government on healthcare policies and strategies, the HA also has the responsibility to promote, assist and take part in research relating to hospital services (Chapter 113, Section 4(f) (ii) of the laws of Hong Kong).

2. Local Regulation on Clinical Studies of Pharmaceutical Products

Clinical studies of pharmaceutical products are regulated in Hong Kong under the Pharmacy and Poisons Regulations (Chapter 138A Regulation 36B of the laws of Hong Kong). For the purpose of regulatory compliance, a Certificate for Clinical Trial ("CTC") shall be obtained before initiation of clinical study of pharmaceutical product.

3. Acceptance of Sponsorship for Clinical Research

The HA is a statutory independent organisation funded by public revenue and has a set of policies and guidelines on acceptance of sponsorship in order to manage and avoid possible conflict of interest situations. Please take note of the "Key Points to Note for Acceptance of Sponsorship" if sponsorship to the clinical research is expected.
http://www.ha.org.hk/haho/ho/hrd/key_letter_Eng1.pdf

☑ Acknowledge of the instruction

Clinical Research Ethics Review Application Form

Fields mark with asterisk (*) are mandatory fields

IRB/ REC Reference No. (For Office Use): UW 20-810

PART I: OUTLINE OF APPLICATION

1. Name of Study

1.1 Scientific Title (<500 characters)*

Association of aspirin with incident lung carcinoma in Chronic Obstructive Pulmonary Disease patients: a population based cohort study

1.1.1 Research protocol number:

1.2 Short Title (for lay public / easy quote)*

The use of Aspirin and the risk of lung cancer

1.3 Key Words (for searching purpose, e.g. disease name, drug name, etc.)*

lung cancer, aspirin, cohort

2. Applicant (Principal Investigator)

2.1 Title * (e.g. Mr, Mrs,Ms, Miss, Dr): Dr   Surname *: Yiu   First name *: Kai Hang

Name in Chinese:

2.2 Position of the Principal Investigator (PI)

2.2.1 ☐ HA staff
- Position:
- Department/Unit:
- Hospital (1):
- Hospital (2):

☐ Site Coordinator (If the PI is not situated in the applying HA site, it is recommended to assign a qualified HA staff for site coordination.)

2.2.2 ☑ University staff
- Position: Clinical Associate Professor
- Department/School/ Faculty: Department of Medicine
- University: HKU

2.2.3 ☐ HA Employee   ☑ University Employee

2.2.4 ☐ Full-time student   ☐ Part-time student

☐ Undergraduate student   ☐ Post-graduate student

Name of Program:

Department/School /Faculty: Institute:

2.2.4.1 Name of academic supervisor:

Department/School/Faculty | | Institute | |

Supporting document from academica

2.2.4.2 Name of site supervisor

Department/Unit

Hospital

2.3 PI's primary affiliated hospital/institution *

HA	University	Others, specify
QMH	HKU	

2.4 Qualifications and relevant experience (<1,000 characters or attach document)*

ACADEMIC QUALIFICATION

1983 - 1989 St. Joseph Primary School, Hong Kong

1989 - 1992 North Toronto High School, Ontario, Canada

1992 - 1996 St. Joseph College, Hong Kong

1996 - 2001 MBBS
Faculty of Medicine, The University of Hong Kong

2010 - 2011 Research Fellow and PhD student
Leiden University Medical Center, the Netherlands
Topic: Novel non-invasive cardiac imaging: Echocardiography and 320 roles computed tomography

2011 - 2012 MD
The University of Hong Kong

PROFESSIONAL TRAINING

07/2001 - 12/2001 Internship
Department of Medicine, Queen Mary Hospital

01/2002 - 06/2002 Internship
Department of Surgery, Queen Mary Hospital

07/2002 - 06/2005 Resident (Basic physician training)
Department of Medicine, Queen Mary Hospital

07/2005 - 06/2008 Resident (Higher physician training in Cardiology)
Division of Cardiology, Queen Mary Hospital

07/2008 - 12/2009 Resident Specialist in Cardiology
Division of Cardiology, Queen Mary Hospital

2.5 Phone number

2.6 Fax number

2.7 E-mail address*
(<500 characters)

PI: khkyiu@hku.hk
Delegates: wongpf@hku.hk
FollowUpUsers: wongpf@hku.hk

(Please fill in all relevant email addresses of the PI, site-coordinators and other relevant members that are responsible to the communication related to the clinical research and ethics application.)

2.8 Mailing address*

Room1929C 19/F Block K Queen Mary Hospital Pok Fu Lam Hong Kong

3. Other investigators

(If the PI is not situated in the applying HA site, it is recommended to assign a qualified HA staff for site coordination.Please also specify if there is a Lead PI in addition to the PI.)

No.	Title	Surname	First name	Email	Relevant Qualifications	Department	Responsibility	HA Site	Institution	Others, Specify

4. Study Site(s)

4.1 Is this a local or international trial? * — Local

4.2 Will the study be conducted in HA hospitals/institutions?* — ☑ Yes ☐ No

4.2.1 Is there a plan to involve more than one HA site? — ☐ Yes ☑ No ☐ Unknown

4.3 Study sites

4.3.1 Applying sites in HA

Cluster	Hospital	Department	Other sites, specify
HKWC	QMH	Learning & Information Centre	

4.3.2 Collaborating site(s) in HA

Cluster	Hospital	Department	Other sites, specify

4.3.3 Study at sites out-of-HA

Country/City	Hospital	Department	Other sites, specify

5. Parallel Ethics Review for Cross-cluster Study

5.1 Has the protocol been reviewed by another Cluster REC/IRB? — ☐ Yes ☑ No/Unknown

5.1.1 What is REC/IRB decision?

(Please attach the supporting document in Part VI, e.g. approval letter)

6. Timetable

6.1 Proposed study start date — 08/12/2020

6.2 Proposed study end date or date of last follow-up of all recruited subjects, whichever is later — 31/12/2021

6.3 Tentative final report date to Cluster REC — 30/06/2022

7. Brief Summary of Study*

(< 6,000 characters, use language that can be understood by laypersons. Technical terms are not recommended and no referral to protocols/other documents is allowed)

We wish to identify all patients with chronic obstructive pulmonary disease (COPD) who attended HA between the year 2005 and 2018 through the clinical data analysis and reporting system (CDARS). All patients are deidentified in the system. We will subsequently look for aspirin use and look for any association with outcomes of COPD, particular lung cancer and risk of bleeding.

8. Major Ethical Issues*

(< 6,000 characters, use language that can be understood by laypersons. Technical terms are not recommended and no referral to protocols/other documents is allowed)

There are no major ethical issues as we will be relying on the clinical data analysis and reporting system (CDARS), where all pateints are deidentified and represented by a reference key and thus the information is not traceable to any patients.

PART II: STUDY DETAILS (No referral to protocols/other documents is allowed)

9. Scientific basis

IRB/ REC Reference No. (For Office Use): UW 20-810

9.1 Background, current evidence and key references* (< 30,000 characters)

Lung carcinoma is the most common cause of malignancy worldwide with an estimated incidence of 2.1 million in 2018, resulting in 1.8 million deaths.1 Cigarette users, of which there are currently 1 billion worldwide, are especially at risk. In addition, chronic obstructive pulmonary disease (COPD), a progressive airway disease caused by cigarette smoking, is by far the most common comorbidity in patients with lung carcinoma, with a prevalence ranging from 30% to 70%.2 Among smokers, the risk of lung carcinoma development is higher in COPD patients than in those without COPD, suggesting that there may be common pathogenic mechanisms between these two entities.3 Given the lack of curative interventions for COPD, preventative strategies for lung carcinoma in COPD patients is urgently needed.
Prior studies have suggested that aspirin may prevent acute exacerbations, reduce hospitalizations and improve lung function as well as survival in patients with COPD.4,5

1. Bray F, Ferlay J, Soerjomataram I, Siegel RL, Torre LA, Jemal A. Global cancer statistics 2018: GLOBOCAN estimates of incidence and mortality worldwide for 36 cancers in 185 countries. CA: A Cancer Journal for Clinicians. 2018;68(6):394-424.
2. Young RP, Hopkins RJ, Christmas T, Black PN, Metcalf P, Gamble GD. COPD prevalence is increased in lung cancer, independent of age, sex and smoking history. Eur Respir J. 2009;34:380-386.
3. Young RP, Hopkins RJ. Link between COPD and lung cance. Respir Med. 2010;104:758-759.
4. Fawzy A, Putcha N, Aaron CP, et al. Aspirin Use and Respiratory Morbidity in COPD: A Propensity Score-Matched Analysis in Subpopulations and Intermediate Outcome Measures in COPD Study. Chest. 2019;155:519-527.
5. Ekstrom MP, Hermansson AB, Strom KE. Effects of cardiovascular drugs on mortality in severe chronic obstructive pulmonary disease. Am J Respir Crit Care Med. 2013;187:715-720.

9.2 Aim of study* (< 30,000 characters)

To see if there are associations between aspirin use and incident lung cancer in COPD patients.

9.3 Hypothesis (e.g. Compared to x control, y intervention leads to a greater rate of z outcome)* (< 30,000 characters)

Aspirin use among COPD patients are associated with lower incidence of lung carcinoma incidence, but likely results in a higher risk of bleeding.

9.4 Outcome measure(s)

9.4.1 Primary outcome(s)* (< 30,000 characters)

The primary outcome is that of lung carcinoma, identified from the clinical information database through diagnostic codings in accordance with the international classification of diseases, version 9.

9.4.2 Secondary outcome(s) (< 30,000 characters)

The primary outcome is that of episodes of bleeding, identified from the clinical information database through diagnostic codings in accordance with the international classification of diseases, version 9.

9.5 In what way will the research contribute to knowledge or healthcare development?* (< 30,000 characters)

COPD currently has no curative interventions, the ability to reduce lung cancer incidence would prove to be an effective intervention among patients with chronic obstructive pulmonary disease.

10. Study subjects

10.1 Inclusion criteria* (< 30,000 characters)

All patients aged 18 or above who attended any HA facilities between 2005 and 2019 diagnosed with chronic obstructive pulmonary disease.

10.2 Exclusion criteria* (< 30,000 characters)

All patients, who, prior to the diangosis of COPD, had taken aspirin on a daily basis, was diagnosed with lung cancer, had excisional lung procedures, or have a history of human immunodeficiency virus. Additionally, all patients who survived less than 90 days after diagnosis of COPD will be excluded.

10.3 Sample-size and rationale for calculation*

Sample size*(< 600 characters)	Sample size calculation is not required as this is a population based study, however, we estimate that over the duration of the study there should be around 90000 cases of COPD patients.
based on the following rationale*(< 30,000 characters)	Chan et al (2018), in Comorbidities, mortality, and management of chronic obstructive pulmonary disease patients who required admissions to public hospitals in Hong Kong - computerized data collection and analysis, noted that there are 9776 patients identified from CDARS in 2012. Thus, since our duration of interest is an order of magnitude larger, the cohort size should be an order of magnitude larger

10.4 Number of subjects to be recruited locally in applying site*

0

10.5 How will subject be identified and recruited* (< 30,000 characters)

Subjects are identified via the clinical data reporting and analysis system, a database managed by the hospital authority with all patient information that are deidentified.

11. Ethical Review for Study

11.1 Applicant's Preference ☐ Full Review ☑ Expedited Review

11.2 Justification (< 1,000 characters)

We do not interact with any patients and only identify patient information via a deidentified system such that 1) there is no risk for any involved patients and 2) confidentiality is maintained.

(The Cluster REC/IRB has full authority to decide the type of ethical review to be conducted)

12. Risk Assessment whether Expedited Review is suitable

		Yes	No
12.1	Will study incur extra clinical intervention(s) to subjects?*	☐	☑
12.2	Will study impose additional risk to subjects?*	☐	☑
12.3	Will study raise sensitive / important privacy concerns?*	☐	☑
12.4	Will the study involve the following vulnerable subjects?		
12.4.1	Children or adolescent (of less than 18-year-old)*	☐	☑
12.4.2	Illiterates*	☐	☑
12.4.3	Mentally incapacitated persons*	☐	☑
12.4.4	Impoverished persons*	☐	☑
12.4.5	Ethnic minority groups*	☐	☑

12.4.6 Patients in emergency conditions* ☐ ☑

12.4.7 Prisoners* ☐ ☑

12.4.8 Subordinates or students of investigators* ☐ ☑

12.4.9 Others* ☐ ☑

Specify

12.5 Are there any special precautions to protect the interest of vulnerable subjects? (<1,000 characters)

12.6 Will study impose potential hazard to clinical staff?* ☐ Yes ☑ No

12.6.1 Control measures to protect the clinical staff (<1,000 characters)

13. Study Design and Methodology

13.1 Study design* Retrospective and Cohort study (Compare different cohorts)

If others, specify

13.1.1 Control* Concurrent control (cohort)

13.1.2 Group assignment* Parallel

13.2 Disease group (choose the most appropriate one)* Respiratory system

13.2.1 Key conditions under study (e.g. Asthma; DM; etc.)

Chronic obstructive pulmonary disease; Lung cancer

14. Methods of Data Analysis* (<30,000 characters)

Inverse probability of treatment weighting was used to balance baseline covariates between aspirin nonusers with new aspirin users. Competing risk regression with Cox proportional-hazard models were performed to estimate the subdistribution hazard ratio (SHR) of lung carcinoma with aspirin and the associated bleeding events.

15. Handling and Storage of Personal Data

15.1 How will the personal data be handled and stored during and after the study?* (<2,000 characters)

All personal data are deidentified as stored in the clinical data reporting and analysis system. They will be deleted after the study.

15.2 Who will be responsible for safekeeping of the personal data during and after the study?* (<2,000 characters)

The investigators, Wong Pui Fai, will be responsible for this.

15.3 Who will have access to the personal data during and after the study?* (<2,000 characters)

All investigators and the principle investigator as previously listed will have access to the deidentified data.

15.4 How long will the personal data be kept after the study?* (<2,000 characters)

The deidentified data will be kept for 3 months after the study.

15.5 Plan arrangement for the personal data after completion of the aforesaid storage period?* (<2,000 characters)

The deidentified data will be deleted.

PART III: STUDY DETAILS (Sections 16 to 19 are applicable for Prospective Study only)

16. Study Article and Arrangements

IRB/ REC Reference No. (For Office Use): UW 20-810

16.1 Study design

16.1.1 How does the procedure/treatment differ from current treatment practice?*

if others, specify

16.1.2 Methods of assignment*

16.1.3 Degree of masking*

16.1.4 Phase of study*

16.2 Study article

16.2.1 Is there any study article?* ☐Yes ☐No

16.2.2 Study article details

Article	Type	Name	Duration of exposure	Dosage	Route of administration	Was it produced under GMP?	Others, specify

Control

No.	Type	Name	Duration of exposure	Dosage	Route of administration	Was it produced under GMP?	Others, specify

16.2.3 Study article licence registration

			Same indication registration			Other indication registration		
Article	Type	Name	HongKong	In Oversea	Overseas Country	Hong Kong	In Oversea	Overseas Country

** Overseas Country should be blank when the answer of In Oversea is "No"*

Control

			Same indication registration			Other indication registration		
No.	Type	Name	Hong Kong	In Oversea	Overseas Country	Hong Kong	In Oversea	Overseas Country

** Overseas Country should be blank when the answer of In Oversea is "No"*

16.2.4 Will a Certificate for Clinical Trial ("CTC") be obtained for the Study? ☐Yes ☐No

If no, justification (<500 characters)

16.3 Will the study register in public domain trial registry, e.g. ClinicalTrials.gov?

☐Yes ☐No/Not Applicable ☐Unknown

If yes, Responsible party/parties for registration, e.g. sponsor, PI

If no, justification (<500 characters)

16.4 Is there a plan to apply for a clinical trial approval from National Medical Products Administration (NMPA) in the People's Republic of China? * ☐ Yes ☐ No ☐ N/A

16.5 Has a Phase I study been done?* ☐ Yes ☐ No

16.6 Number of extra visits / admission on top of usual care*

16.7 Will any of the study interventions / procedures be performed by persons other than the investigators?* ☐ Yes ☐ No

If yes, by whom

If yes, where

16.8 Will biological samples be stored for future use?* ☐ Yes ☐ No

16.8.1 What is the purpose to store the sample? (<1,000 characters)

16.8.2 State the nature of the sample (<500 characters)

16.8.3 Anticipate duration of storage

16.8.4 Will the samples be sent / stored outside Hong Kong? ☐ Yes ☐ No

16.8.5 Will consent[1] for future specified usage of the biological samples be obtained from the subjects? ☐ Yes ☐ No

16.8.5.1 When will the consent be obtained?

Justification for consent to be obtained prior to the specific future usage of the sample (<500 characters)

16.8.5.2 Where will the consent be stated?

16.8.5.3 Justification for no consent to be obtained (<1,000 characters)

17. Potential Risk Arising from Study

17.1 Induce discomfort or distress* ☐Yes ☐No

17.2 More invasive than the usual management * ☐Yes ☐No

17.3 Increase physical or psychological risk* ☐Yes ☐No

17.4 Involve a potential toxin, mutagen or teratogen* ☐Yes ☐No

17.5 Involve radiation or radioactive substance* ☐Yes ☐No

17.6 Incur other hazards* ☐Yes ☐No

17.7 If yes to any of the above, provide details (<1,000 characters)

17.8 Significant difference(s) from usual management* (<1,000 characters)

18. Anticipate Benefits to Study Subjects* (<1,000 characters)

19. Research Subject Protection

19.1 Will the subjects be provided with a card indicating their participation in study and means of urgent contact?* ☐Yes ☐No

If no, state how the research subjects can be identified in case of emergency (<1,000 characters)

19.2 Does the protocol state compliance with the ICH-GCP?* ☐Yes ☐No

If no, justification (<1,000 characters)

20. Information and Consent

(The informed consent should state HKU IRB / Hong Kong West Cluster REC as one of the authorized parties to access the subjects' records related to the study for ethics review purpose.)

20.1 Methodology of obtaining consent*

No (subject to wavier by REC)

20.1.1 State reasons if not use written consent (<1,000 characters)

20.1.2 Justification for applying to waive the consent requirement (<1,000 characters)

This is a rerospective study using the electronic health care database, which all patients are anyomised.

20.2 Who will carry out the informed consent process with the subject? (can select more than one option)

☐ Principal investigators
☐ Other investigators
☐ Research assistant
☐ Others, specify:

20.3 Will an interpreter be available when required? ☐ Yes ☐ No

If no, justification

20.4 In obtaining informed consent from subjects, what is the minimal time given to a subject to consider after explanation has been given?

20.5 If subjects are incompetent in giving consent, what would be the arrangement? (<500 characters)

21. Data and Safety Monitoring

21.1 Will an independent committee review data and safety of study?* ☐ Yes ☑ No

If no, justification (<500 characters)

All patient information is deidentified as obtained from the clinical information database, and there will be no interactions with any patients,

[1]Obtaining consent from subjects for future use of the biological samples is compulsory.

PART IV: BUDGET AND USE OF RESOURCES

IRB/ REC Reference No. (For Office Use): UW 20-810

22. Source of Funding

22.1 Commercial* ☐ Yes ☑ No

22.1.1 Sponsored trial ☐ Yes ☑ No

Specify the source of funding:

No.	Name of Sponsor / donating body

22.2 Non-commercial* ☐ Yes ☑ No

22.2.1 Sponsored trial ☐ Yes ☑ No

Specify the source of funding:

No.	Type of funding	Name of Sponsor / donating body

22.3 Other funding sources (e.g. personal funded study), provide name(s) and background information (<1,000 characters or attach document)

No other funding sources.

23. Resources Implication and Conflict of interest

23.1 Will this study consume HA resources?* ☑ Yes ☐ No

23.1.1 If yes, provide details (<1,000 characters)

It will utilise the clinical data analysis and reporting system maintained by the hospital authority.

23.2 Will the study involve HA patients?* ☑ Yes ☐ No

If yes, estimate the number of patients planned to involved in the study? 90000 patients

If no, specify the role of HA in the study (<500 characters)

23.3 If HA resources is required, how will this affect the HA services of other patients with competing needs? (<500 characters)

It will not affect HA services of other patients because all information is retrieved from a digital database. It will also no affect any patients.

23.4 Will the study site (hospital) receive reimbursement for the study? ☐ Yes ☑ No

If yes, state the format of reimbursement (<1,000 characters)

If no, state the reason(s) (<1,000 characters)

The study sites will not be affected by the study in any manner, since information retrieval is digital and requires no physical interactions.

23.5 Is there a non-monetary sponsorship?* ☐ Yes ☑ No

23.5.1 Type of sponsorship

☐ Drug ☐ Consumable ☐ Equipment ☐ Research assistant

☐ Others, specify

24. Financial Costs and Payment to Subjects

24.1 Will the subjects be charged for the study article/service?* ☐ Yes ☑ No ☐ N/A

If yes, state the financial arrangement (<500 characters)

24.2 Will the study article continue to be available to subjects after the study (if subjects benefited from it) until it is commercially available? ☐ Yes ☑ No

If yes, state the planned financial arrangement (<500 characters)

If no, will there be any impact on disease management of the study subject? ☐ Yes ☑ No

Provide arrangement (<500 characters)

24.3 Does the consent form explain the above arrangement? ☐ Yes ☑ No

24.4 Will subjects receive any material rewards (including payments)?* ☐ Yes ☑ No

24.4.1 Reward nature* ☐ Cash ☐ Others (<200 characters)

24.4.2 Amount of payment (in HK$)

24.4.3 Mode of payment ☐ One-off

☐ By schedule (<500 characters)

25. Research Organization and Indemnity

25.1 The organization / individual responsible for the study* (<500 characters)

Dr. Yiu Kai Hang, in the department of medicine, university of Hong Kong.

25.2 Collaborating parties that jointly take on the responsibilities for the study

Collaborating Party	Name of organization / individual

25.3 Indemnity

25.3.1 For industry sponsored trial, will the sponsor indemnify study related claims? ☐ Yes ☐ No

If no, specify the reason and who will be responsible for the indemnity (<500 characters)

Is the indemnity agreement based on the HA approved form? ☐ Yes ☐ No

25.4 Will an insurance policy be arranged for the study?* ☐ Yes ☑ No

25.4.1 Will the policy be reviewed by HA Legal Service Department? ☐ Yes ☐ No

25.5 Will a Clinical Trial Agreement or other legal agreement/contract(s) be signed with the sponsor/supporting party?*

☐ Yes, with HA ☐ Yes, with University ☑ No

PART V: DECLARATION BY INVESTIGATOR(S)

IRB/ REC Reference No. (For Office Use): UW 20-810

Note: Certain trial information will be passed to a Central Database for risk management purpose and to assist HA's finance controller in sourcing insurance coverage for clinical trial activities

26.1:Scientific Title of Study

Association of aspirin with incident lung carcinoma in Chronic Obstructive Pulmonary Disease patients: a population based cohort study

1. I / We declare that the information supplied is to the best of our knowledge and accurate.
2. I / We declare that the protocol comply with Declaration of Helsinki.
3. I / We agree to uphold the protection of research subjects' right and safety through adherence to local laws, Declaration of Helsinki, institutional policies[2] and whenever applicable, the ICH-GCP.
4. I / We understand that approval by the Cluster REC is subject to regular renewal according to local policy.
5. I / We agree to report to the HKU IRB / Hong Kong West Cluster REC
   - any planned change(s) to the study, and further agree not to implement any change(s) without receiving prior approval, except to eliminate immediate hazard to research subjects or when the change(s) involve only logistical or administrative issues.
   - any fatal events in applying site within the specific time according to the Standard Operating Procedures of the Cluster REC while pending investigation, and any serious adverse events in applying site (with an extended report) preferably within seven days but not later than 15 days (from the day it was made known to me / us).
   - any new information on the project that adversely influences the risk/benefit ratio.
   - progress report(s) (as requested by the Cluster REC) and a final report (after completion of study).
6. I / We agree to keep all study documents for a period of at least three years after study closure.
7. I / We agree to maintain adequate records and to make them available for audit / inspection.
8. I / We agree to ensure that all associates, colleagues, and employees assisting in the conduct of the study are informed about their obligations in meeting the above commitments.

26.2:Signed by Principal Investigator and Other Investigators

Role	Title	First Name	Surname	Position	Responsibility for clinical oversight	Signature	Date (DD/MM/YYYY)
Principal investigator	Dr	Kai Hang	Yiu	Clinical Associate Professor	Y		

26.3:For Student Project

Role	Name	Position	Responsibility For clinical oversight	Signature	Date (DD/MM/YYYY)

[2]HA Guide on Research Ethics (for Study Site & Research Ethics Committee) and Investigator's Code of Practice; HA Clinical Data Policy Manual; and other prevailing HA policies.

26.4:Endorsement by COS or Authorised Representative[3] for

Scientific Title of Study

Association of aspirin with incident lung carcinoma in Chronic Obstructive Pulmonary Disease patients: a population based cohort study

1. I endorse the application and authorise the captioned study to be undertaken in my department upon approval by the Cluster REC/IRB.
2. I am of the opinion that the investigator(s) within my department/unit are appropriately qualified within the disease / therapeutic area involved, and are capable of undertaking this study in terms of their workload and time available, and that the study site(s) under my supervision have access to adequate facilities and support for the research to be conducted in a safe manner.

Signature	Name	Email	Post	Department	Hospital	Date

[3]Should be signed by another suitable senior staff (e.g. HCE or his/her designate) if the COS is the Applicant for the study

26.5:Endorsement by Head of Department[4] Contributing to the Research

Scientific Title of Study

Association of aspirin with incident lung carcinoma in Chronic Obstructive Pulmonary Disease patients: a population based cohort study

1. I endorse the application and authorise the captioned study to be undertaken in my department upon approval by the Cluster REC/IRB.
2. I am of the opinion that the investigator(s) within my department/unit are appropriately qualified within the disease / therapeutic area involved, and are capable of undertaking this study in terms of their workload and time available, and that the study site(s) under my supervision have access to adequate facilities and support for the research to be conducted in a safe manner.

Signature	Name	Email	Post	Dept/School/Faculty	Institution	Date

[4]Should be signed by another suitable senior staff (e.g. Acting Head / Senior Member in the Department) if the Head of Department is the Applicant for the study

26.6:Endorsement by COS(s) or Head(s) of Other Department(s)[5] Contributing to the Research

Scientific Title of Study

Association of aspirin with incident lung carcinoma in Chronic Obstructive Pulmonary Disease patients: a population based cohort study

I support the captioned study and verify that the workload to be incurred will not interfere with the department's service priority.

Signature	Name	Email	Position	Department	Hospital	Date

[5]If the study involved other departments, it is the Applicant's obligation to inform and obtain agreement with the COS(s) or Head(s) of the Department(s).

IRB/REC Name

Clinical Study Categorization Form

IRB/ REC Reference No. (For Office Use): UW 20-810

Note to Investigator

Please complete the following Clinical Study Categorization Form and submit the Form together with each application for research ethics review. Upon receipt of an application, the Secretariat will verify the information on the form and arrange for appropriate initial review through Full Review, Expedited Review or Full Review by Phase 1 Panel.

Risk Group	No.	Risk Factors	Yes	No
Human Subjects	1	Recruitment of human subjects	N	Y
	13	------------------------------END--------------------------------		

Application Log

Note to Investigator

This Application Log shows all the updated information, which are extracted from your Research Ethics Review Application Form and your subsequent submissions for REC/IRB's review and approval.

General Information

Work Order Number:	WO-120534
Submission Reference Number:	HKWC-2020-0877
IRB/ REC Reference Number:	UW 20-810
Initial Application Submission Date :	04/12/2020
Initial Application Review Type :	
Initial Application Approval Date :	
Approval Expiry Date:	
Proposed Study Start Date:	08/12/2020
Proposed Study End Date:	31/12/2021
Actual Study Start Date:	
Actual Study End Date:	
Initial Study Subject Recruitment Date:	
CTC Expiry Date:	
CTI Expiry Date:	
Latest Progress Report Submission Date:	
Final Report Submission Date:	
Terminatation Date:	
Termination Reason:	

Status History

Date	Task	User	From	To	Open Form
18/11/2020	Initial Application Approval	Pui Fai Wong	New	Draft	Click Here
04/12/2020	Initial Application Approval	Pui Fai Wong	Draft	Submitted	Click Here
04/12/2020	Initial Application Approval	Clara LEUNG	Submitted	Confirmed	Click Here
07/12/2020	Initial Application Approval	Chris YIP	Confirmed	Returned	Click Here

Approval History

Date	Task	Application Status	Review Due Date	Review Type	Decision	Decision Date	Decision Reason
07/12/2020	Initial Application Approval	Returned					

Document Log

General Information

Work Order Number:	WO-120534
Submission Reference Number:	HKWC-2020-0877
IRB/ REC Reference Number:	UW 20-810

Uploaded Documents

Date	User	Document Type	Document Name	Suggested Print Name	Upload Times
19/11/2020	Pui Fai Wong	Research Protocol	Research Protocol.docx		1
19/11/2020	Pui Fai Wong	Curriculum Vitae (CV) from Principal Investigator	YKH CV (brief).docx		1

Application Management Team Member Form

Note to Investigator
Please complete the following Application Management Team Member Form before opening a new Application Form. Upon submission of the Form, each of the team members (PI, Delegates, Application Follow-up Users) will receive a notifying email to start contribute to this Application.

Principal Investigator :

Email	Name	Existing Account	Please sign up
khkyiu@hku.hk	Kai Hang Yiu	Y	

Assign Principal Investigator who will be responsible for the Application.

Delegates :

Email	Name	Existing Account	Please sign up
wongpf@hku.hk	Pui Fai Wong	Y	

Please assign Delegates who will help manage and edit the application forms before research ethics approval.

Application Follow Up Users :

Email	Name	Existing Account	Please sign up
wongpf@hku.hk	Pui Fai Wong	Y	

Please assign Follow-up Users who will help manage and follow up the post-approval activities.

Application Review Arrangement

Submission Ref. No. :	
IRB/REC Ref. No. :	
Type of Review :	
Review Due Date :	
Review Meeting Date :	
Review Panel :	

Panel Member :

Name	Gender	Institution	Department	Post	Chairman	Role	Independent

Member Review Progress :

Name	Status	Last Update	Send to Reviewer

Meeting Attendance :

Name	Decision	Attend the meeting?	Conflict of Interest?	Relationship

Decision :	
Decision Date :	
Approval Expiry Date :	

Viewed by Applicant

Comment History :

Date	Name	Comment	To
07/12/2020	Chris YIP	Return to PI for updating of information.	Applicant

Please provide your comment if needed
